# Unlocking p-coumaric acid’s potential against biofilm-forming extensively drug-resistant *Acinetobacter baumannii*: in vitro and in vivo study

**DOI:** 10.1186/s12941-026-00859-2

**Published:** 2026-04-11

**Authors:** Noha A. Attia, Ahmed A. Abdelaziz, Fatma I. Sonbol, Amal M. Abo-Kamar, Lamiaa A. Al-Madboly

**Affiliations:** Department of Microbiology and Immunology, Faculty of Pharmacy, Tanta University, Tanta, Egypt

**Keywords:** *Acinetobacter baumannii*, Extensively drug-resistant, Biofilm, p-Coumaric acid, Resistance modulation

## Abstract

**Background:**

The global rise of extensively drug-resistant *Acinetobacter baumannii*, particularly biofilm-forming strains, has drastically limited treatment options and created an urgent need for novel therapies to restore antibiotic efficacy. This study explored p-coumaric acid (*p*-CA) as a potential dual-action agent to combat biofilm-associated XDR *Acinetobacter baumannii* infections and restore imipenem efficacy.

**Methods:**

Among 100 clinical *Acinetobacter baumannii* isolates, 32 were identified as XDR and exhibited resistance to imipenem. The antimicrobial and antibiofilm efficacy of *p*-CA was systematically evaluated through comprehensive in vitro assays and an in vivo rat infection model. Minimum inhibitory concentrations were determined via the broth microdilution method, and the potential modulation effect on imipenem efficacy was investigated. To assess biofilm inhibition and disruption, quantitative analyses were performed using the crystal violet staining technique, complemented by evaluating its impact on the bacterial cell surface hydrophobicity and exopolysaccharide production. Biofilm structural changes were analyzed via light, scanning electron, and confocal laser scanning microscopy. Additionally, the expression levels of key biofilm-associated genes were quantified via quantitative reverse transcription PCR.

**Results:**

The *p*-CA exhibited potent antimicrobial activity against the tested isolates (MIC: 512 µg/mL) and synergized with imipenem, reducing its MIC by 512-fold. At subinhibitory concentrations (¼–½ MIC), it inhibited biofilm formation (66.2–80.5%, *p* < 0.05) and disrupted pre-formed biofilms (45.8–71.3%, *p* < 0.05), likely via altered cell surface hydrophobicity and reduced EPS production. Microscopic imaging corroborated these findings, revealing substantial structural degradation of biofilms upon treatment. At the molecular level, *p*-CA significantly downregulated (*p* < 0.05) the key biofilm-associated genes (*abaI*, *bfmR*, *bap*, *csuE*, and *pgaB*), as quantified by RT-qPCR. In vivo, the *p*-CA/imipenem combination significantly enhanced the survival rates (100%, *p* < 0.05) and reduced the lung bacterial burden (*p* < 0.001). Histopathological examination showed near-complete restoration of alveolar architecture by 72 h post-treatment in the combination therapy group.

**Conclusion:**

These findings position *p*-CA as a promising dual-action adjuvant against XDR *Acinetobacer baumannii* infections, particularly in biofilm-associated contexts. It combines direct antimicrobial activity, biofilm disruption, and synergy with imipenem to address critical treatment gaps.

**Supplementary Information:**

The online version contains supplementary material available at 10.1186/s12941-026-00859-2.

## Introduction

*Acinetobacter baumannii* (*A. baumannii*) is a major cause of hospital-acquired infections and has gained global attention due to its rapid adaptation and persistence in healthcare environments. The World Health Organization classifies *A. baumannii* as a “Priority 1, Critical” threat within the ESKAPE group, which collectively accounts for most treatment-refractory nosocomial infections worldwide [1]. Infection risk is amplified by prolonged hospitalization, intensive care admission, invasive procedures, and underlying comorbidities. Cancer patients are particularly vulnerable because of immune suppression related to malignancy and anticancer therapies, in addition to antibiotic exposure, mucosal injury, and repeated hospitalizations. Importantly, the organism’s strong capacity to colonize abiotic and biotic surfaces and form biofilms promotes persistence, transmission, and therapeutic failure, particularly in infections involving indwelling devices and compromised host defenses [2].

The clinical management of *A. baumannii* is increasingly complicated by the emergence of multidrug-resistant (MDR) and extensively drug-resistant (XDR) strains. MDR refers to non-susceptibility to at least one agent in three or more antimicrobial categories, whereas XDR indicates non-susceptibility to all but two or fewer categories, leaving extremely limited therapeutic options [3]. Carbapenems, historically among the most reliable agents, are increasingly ineffective, with resistance rates exceeding 70% in many regions, thereby necessitating alternative interventions [4]. Treatment is further undermined by biofilm formation, which reduces antibiotic penetration, enhances tolerance, and facilitates horizontal gene transfer [5]. Biofilm development in *A. baumannii* is governed by quorum sensing and biofilm-associated regulatory and structural determinants, including *abaI*,* bfmR*,* bap*,* csuE*, and *pgaB*, which collectively mediate surface attachment, intercellular aggregation, extracellular polymeric substance production, and maturation [6]. Consequently, effective management of XDR *A. baumannii* (XDRAb) requires strategies that not only inhibit growth but also attenuate biofilm formation and destabilize established biofilms, ideally as adjuncts to conventional antibiotics [7].

Natural products, particularly plant-derived secondary metabolites, have emerged as promising low-toxicity antimicrobial and antibiofilm candidates, either alone or as antibiotic adjuvants. Among these, cinnamic acid derivatives have demonstrated broad pharmacological properties, including antibacterial and antibiofilm effects [8]. Para-coumaric acid (*p*-CA), a phenolic cinnamic acid, has been reported to impair bacterial viability by disrupting membrane integrity, inducing cellular leakage, and altering morphology, and may also interfere with DNA structure [9]. Importantly, *p*-CA has shown antibiofilm potential through suppression of quorum sensing and biofilm-related gene expression [8]. However, its dual antibacterial and antibiofilm efficacy against biofilm-forming XDRAb, particularly in infections affecting cancer patients, remains insufficiently characterized. To address this gap, we conducted a comprehensive study to assess the antibacterial and antibiofilm activities of *p*-CA against imipenem-resistant XDRAb (IXDRAb) isolates recovered from cancer patients. Specifically, we examined the ability of *p*-CA to (1) inhibit bacterial growth and biofilm formation, (2) enhance the efficacy of imipenem, a carbapenem antibiotic, and (3) disrupt preformed biofilms and reduce EPS production. Additionally, we assessed the in vivo efficacy of *p*-CA in a rat model of XDR *A. baumannii* infection. This study contributes to the growing body of evidence supporting the use of natural compounds as adjuvants to conventional antibiotics in combating antimicrobial resistance. By simultaneously targeting antibiotic resistance and biofilm integrity, *p*-CA represents a promising strategy for overcoming the dual challenges IXDRAb poses.

## Methods

### Bacterial isolates

100 clinical *A. baumannii* isolates were collected from patients hospitalized in the Oncology Departments of Tanta University Hospitals (August 2020–August 2022). Samples included sputum, endotracheal aspirates, wound exudates, abscesses, and blood. Preliminary identification relied on colony morphology, Gram staining, and standard biochemical reactions (oxidase, catalase, indole, and carbohydrate fermentation). Species-level confirmation was performed by PCR detection of the intrinsic *bla*_OXA−51−like_ gene [10]. *Acinetobacter baumannii* ATCC 19606 served as the positive control. Isolates were preserved at − 80 °C in nutrient broth supplemented with 20% glycerol [11].

### Antimicrobial susceptibility testing

Antimicrobial susceptibility testing (AST) followed Clinical and Laboratory Standards Institute (CLSI, 2020) guidelines [12]. The susceptibility profiles were evaluated against twenty antimicrobial agents representing nine distinct antibiotic classes. The Kirby–Bauer technique on Mueller–Hinton agar (MHA) (Hi-Media, India) evaluated susceptibility to 19 antibiotics (Oxoid, England). Colistin (CT) (Sigma-Aldrich, United States) susceptibility was determined separately using the broth microdilution, as described previously [13, 14]. Multidrug-resistant (MDR) and extensively drug-resistant (XDR) phenotypes were assigned according to Magiorakos et al. [3]. The multiple antibiotic resistance (MAR) index was calculated via [15] :

$$\begin{array}{l}MAR~index~for~isolates\\ = \frac{{Number~of~antibiotics~to~which~the~isolate~was~resistant}}{{Total~number~of~antibiotics~to~which~the~isolate~was~exposed}}\end{array}$$

### Antibacterial activity of p-coumaric acid

A working solution of 2 mg/mL p-coumaric acid (*p*-CA) (Sigma-Aldrich, United States) was prepared in 0.5% dimethylsulfoxide (DMSO) [16]. Its MICs were determined by broth microdilution in cation-adjusted Mueller-Hinton broth (CAMHB; Hi-Media, India) using resazurin as a viability indicator [14, 17].

### Evaluation of the antibiotic resistance-modulating activity of p-coumaric acid

Imipenem (Sigma-Aldrich, United States) was selected as the test antibiotic to evaluate its combination with *p*-CA in 32 imipenem-resistant extensively drug-resistant *A. baumannii* (IXDRAb) isolates. Imipenem MICs alone and in combination with subinhibitory concentrations of *p*-CA (¼ MIC and ½ MIC) were determined via the broth microdilution method in CAMHB [12]. The ability of *p*-CA to lower the MIC of imipenem was quantified as modulation factor (MF) [13].

### Screening for biofilm formation

Biofilm formation was screened using the microtiter plate crystal violet assay as previously described [18], with full procedural details provided in Supplementary Methods S1. Isolates were classified as non-, weak, moderate, or strong biofilm producers using the cut-off OD approach [13].

### Growth kinetics assay

Five representative isolates of each biofilm category and ATCC 19606 were cultured in TSB in 96-well plates. OD₆₀₀ was measured every 4 h for 48 h. Parallel wells included sub-MIC *p*-CA (¼, ½ MIC) [13].

### Antibiofilm activity of p-coumaric acid at subinhibitory concentrations

Preventive and therapeutic antibiofilm activities of *p*-CA (¼ or ½ MIC) were assessed using early-adhesion and mature-biofilm models, respectively [19, 20]. The calculations were performed using established formula [21], as detailed in Supplementary Methods S2.

### The effect of p-coumaric acid on the metabolic activity of biofilm bacteria

The MTT (3-(4,5-dimethylthiazol-2-yl)-2,5-diphenyltetrazolium bromide) assay was used to assess the metabolic activity of biofilm-embedded bacteria treated with *p*-CA based on reduction of MTT to formazan by viable cells (Supplementary Methods S3) [22].

### Exopolysaccharide quantification and cell surface hydrophobicity

Exopolysaccharide (EPS) was quantified using the phenol–sulfuric acid method [23], and cell surface hydrophobicity was evaluated by the microbial adhesion to hydrocarbons (MATH) assay [24, 25]. Detailed procedures and calculations are provided in Supplementary Methods S4 and S5, respectively.

### Microscopy analysis of antibiofilm activity of p-coumaric acid

A representative strong biofilm-forming IXDRAb isolate (**Ac88**) was selected to visualize the impact of *p*-CA on mature biofilms using light microscopy, scanning electron microscopy, and confocal laser scanning microscopy, following previously reported protocols [13, 26]. Complete experimental procedures are described in Supplementary Methods S6.

### Quantitative real-time PCR analysis of biofilm-associated gene expression

Gene expression analysis of *abaI*,* bfmR*,* bap*,* csuE*, and *pgaB* was performed by RT-qPCR in four strong biofilm-producing IXDRAb isolates cultured with or without *p*-CA (½ MIC, 24 h). *16 S rRNA* served as the internal reference gene [27], and relative expression was calculated using the 2^–ΔΔCT^ method [28]. Primer sequences and RT-qPCR details are provided in Supplementary Methods S7.

### In silico molecular docking study

Molecular docking of *p*-CA against BfmR protein, the response regulator [29], (PDB ID: 5HM6, 10.2210/pdb5HM6/pdb) was performed using MOE 2019.0102 software. Protein hydrogens and charges were optimized using AMBER10: EHT. The BfmR active site was identified using MOE site finder. *p*-CA structure (PubChem, https://pubchem.ncbi.nlm.nih.gov/compound/P-Coumaric-Acid) was docked using triangular matcher placement and London dG scoring, with 2D and 3D interaction diagrams generated for analysis [30, 31].

### In vitro cytotoxicity (MTT) assay

Human skin fibroblast normal cells (National Research Centre, Cairo, Egypt) were treated with serial two-fold *p*-CA dilutions for 48 h. The MTT assay (OD₅₇₀) was performed as previously described, with IC₅₀ calculated from the dose-response curve (Supplementary Methods S8) [32].

### In vivo evaluation in immunocompromised rats

An immunocompromised pneumonia model was established using the strongest biofilm-forming isolate, *A. baumannii* **Ac88** (MAR index 0.9). Forty Male Wistar rats (140–165 g) were transiently immunosuppressed by intraperitoneal cyclophosphamide (150 mg/kg on days − 4 and − 1) before intranasal challenge with *A. baumannii* **Ac88** (10⁸ CFU/mL, 50 µL, day 0) [33, 34]. Successful establishment of infection was verified by Giemsa-stained lung smears obtained from sentinel animals at 0 and 3 h post-inoculation.

Animals were randomly allocated into five groups (*n* = 8): Group 1, normal control; Group 2, infected/untreated; Group 3, infected + imipenem (2 mg/kg); Group 4, infected + *p*-CA (256 mg/kg); Group 5, infected + imipenem + *p*-CA. Treatments were administered intraperitoneally once daily for three days, starting 6 h after inoculation. Rats were monitored for body weight, clinical signs, and survival over seven days. At 72 h, two rats per group were euthanized for blood cell counting and lung harvesting for bacterial load determination and histopathology. For histopathology, lungs were fixed in 10% neutral-buffered formalin, paraffin-embedded, sectioned, and stained with H&E and Giemsa following standard protocols. Liver and spleen tissues were examined using histopathology to assess potential treatment-related toxicity [34, 35].

### Statistical analysis

All the experiments were performed in triplicate, and the results are presented as the means ± standard deviations (SD). Statistical analysis was performed using GraphPad Prism 9. Group comparisons used unpaired *t*-tests or one-way ANOVA with post-hoc testing, and survival curves were analyzed by the log-rank test. Significance was set at *p* < 0.05.

## Results

### Identification, resistance profiles, and biofilm-forming capacities of XDR *Acinetobacter baumannii* isolates

**Fig. 1:**
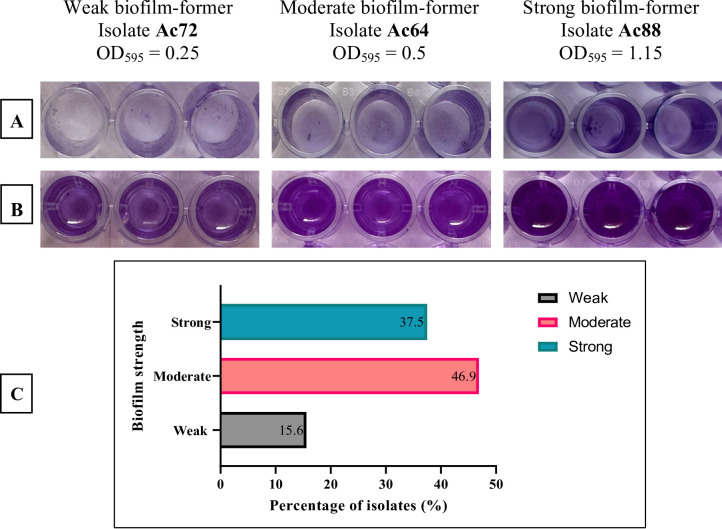
Results of the 96-microtiter plate method for biofilm assay of representative weak, medium, and strong biofilm-producing *Acinetobacter baumannii* isolates. **A** before solubilization of biofilm-containing crystal violet by 33% (v/v) glacial acetic acid. **B** after solubilization of biofilm-containing crystal violet by 33% (v/v) glacial acetic acid. **C** Distribution of biofilm capacities among the tested 32 *Acinetobacter baumannii* isolates, categorized as weak, moderate, or strong producers based on optical density (OD) measurements

Among 100 clinical *A. baumannii* isolates recovered from oncology patients, 32 (32%) were imipenem-resistant extensively drug-resistant (IXDRAb). Species identification was confirmed by PCR detection of *bla*_OXA−51−like_ gene (Supplementary Figure S1). Most IXDRAb isolates were obtained from endotracheal aspirates (18/32, 56.25%), followed by wound, blood, and sputum specimens (Supplementary Table S1). All IXDRAb isolates exhibited multidrug resistance across beta-lactams, cephalosporins, fluoroquinolones, and carbapenems, while colistin remained active against all isolates (Supplementary Table S1, Supplementary Figure S2). Biofilm screening classified 5 isolates (15.6%) as weak, 15 (46.9%) as moderate, and 12 (37.5%) as strong biofilm producers (Fig. 1, Supplementary Table S1).

### Dual antibacterial and resistance-modulating effects of p-coumaric acid

p-Coumaric acid (*p*-CA) showed antibacterial activity against all IXDRAb isolates, with a uniform MIC of 512 µg/mL. At sub-inhibitory concentrations, *p*-CA significantly potentiated imipenem activity in a concentration-dependent manner (Supplementary Figure S3, Supplementary Table S2). At ½ MIC, *p*-CA restored imipenem susceptibility in 31/32 isolates (MIC 0.5–2 µg/mL), while the remaining isolate shifted to intermediate susceptibility (MIC 4 µg/mL), with modulation factors ranging from 32 to 512. At ¼ MIC, imipenem MICs decreased by 2- to 8-fold across isolates, with susceptibility shifting to intermediate in three isolates (MIC 4 µg/mL).

### Impact of sub-inhibitory concentrations of p-coumaric acid on bacterial growth

As shown in Supplementary Figure S4, only minor growth pattern alterations (*p* > 0.05) were observed between the control and *p*-CA-treated isolates, indicating that sub-MIC (¼ MIC and ½ MIC) of *p*-CA generally does not affect the viability of the tested isolates during biofilm development.

### Preventive and therapeutic antibiofilm activities of p-coumaric acid at sub-inhibitory concentrations

#### Preventive antibiofilm assay

**Fig. 2:**
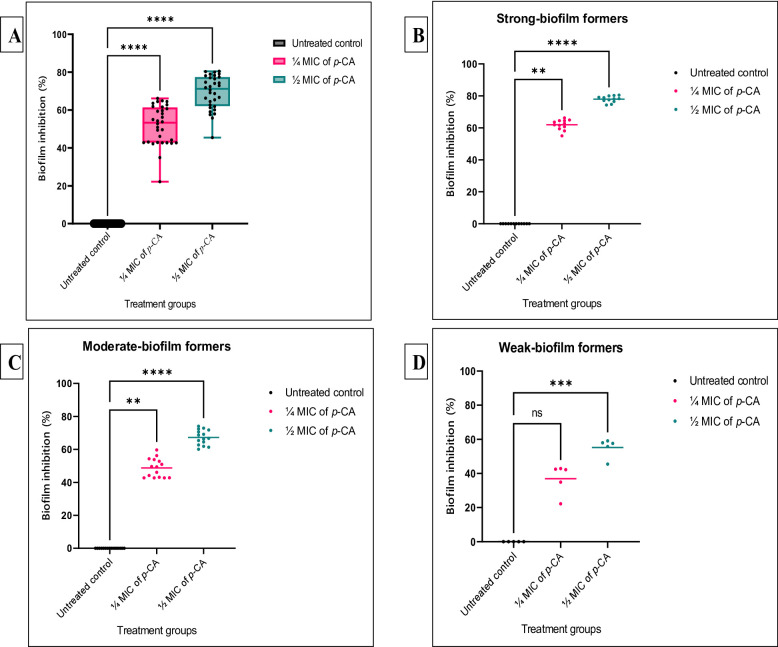
Preventive antibiofilm activity of sub-inhibitory p-coumaric acid (*p*-CA) concentrations (¼ MIC and ½ MIC) against imipenem-resistant XDR *Acinetobacter baumannii* clinical isolates. **A** Distribution of biofilm inhibition percentages among 32 IXDRAb isolates treated with ¼ MIC and ½ MIC of *p*-CA compared with untreated controls, presented as box-and-whisker plots with individual data points (black dots) overlaid. Boxes represent the interquartile range, center lines indicate medians, and whiskers denote minimum and maximum values. **B–D** Biofilm inhibition profiles of isolates stratified according to biofilm-forming capacity: strong (**B**, *n* = 12), moderate (**C**, *n* = 15), and weak (**D**, *n* = 5) biofilm formers. Scatter plots show individual isolate responses, with horizontal lines indicating mean inhibition values. Statistical analysis was performed using one-way ANOVA followed by Dunnett’s post hoc test. ns, not significant (*p* > 0.05); **p < 0.01; ***p < 0.001; ****p < 0.0001 versus untreated control

Sub-inhibitory concentrations (¼ MIC and ½ MIC) of *p*-CA significantly reduced biofilm formation among the tested IXDRAb isolates (*p* < 0.05, Fig. 2A). The most pronounced antibiofilm effect was observed at ½ MIC, achieving an inhibition rate of 80.5%, whereas ¼ MIC achieved 66.2% inhibition compared to untreated controls. Both concentrations differed significantly from the control group as determined by one-way ANOVA followed by Dunnett’s post hoc test. Isolate-specific inhibition percentages for all 32 IXDRAb isolates (Ac55–Ac100) are provided in Supplementary Figure S5. When isolates were stratified according to their biofilm-forming capacity, both ¼ MIC and ½ MIC of *p*-CA significantly reduced biofilm formation in strong and moderate biofilm formers (*p* < 0.05; Fig. 2B–C). In contrast, among weak biofilm formers, only ½ MIC produced a statistically significant reduction (*p* < 0.05; Fig. 2D). Overall, stronger biofilm-producing isolates exhibited greater susceptibility to *p*-CA-mediated biofilm inhibition. Importantly, exposure to sub-MIC concentrations of *p*-CA (¼ and ½ MIC) did not result in significant alterations in bacterial growth kinetics (*p* > 0.05; Supplementary Figure S4), confirming that the observed antibiofilm effects were independent of growth inhibition.

#### Therapeutic antibiofilm assay

**Fig. 3:**
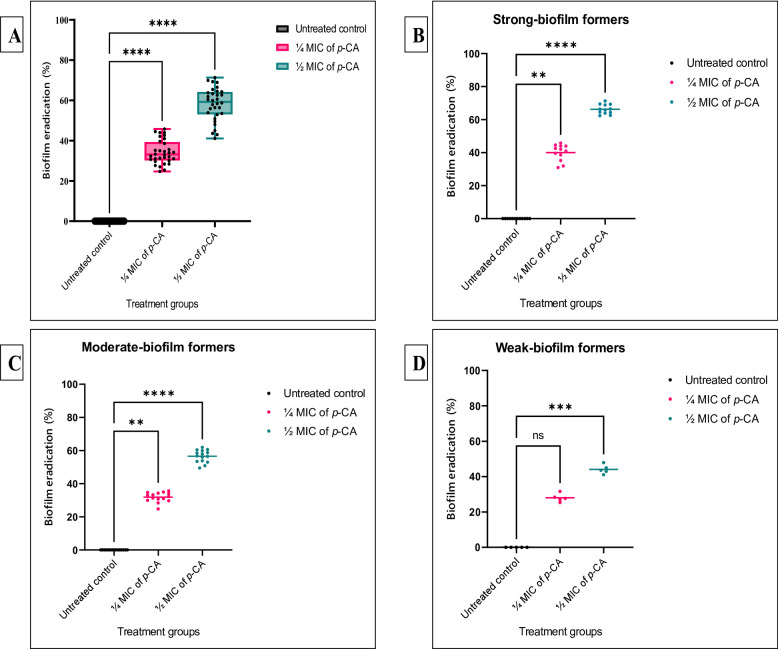
Therapeutic antibiofilm activity of sub-inhibitory p-coumaric acid (*p*-CA) concentrations (¼ MIC and ½ MIC) against imipenem-resistant XDR *Acinetobacter baumannii* clinical isolates (*n* = 32). **A** Box-and-whisker plots illustrate the distribution of mature biofilm eradication percentages following treatment with ¼ MIC and ½ MIC of *p*-CA compared with untreated controls. Boxes represent the interquartile range (25th–75th percentile), horizontal lines denote median values, and whiskers indicate the minimum and maximum observations. Individual data points (black dots) represent biofilm eradication values for each isolate. **B–D** Therapeutic antibiofilm effects of *p*-CA against IXDRAb isolates stratified by biofilm-forming capacity: **B** strong biofilm formers (*n* = 12), **C** moderate biofilm formers (*n* = 15), and **D** weak biofilm formers (*n* = 5). Scatter plots depict individual isolate biofilm eradication percentages at ¼ MIC and ½ MIC, with horizontal bars indicating mean values. Statistical comparisons were performed using one-way ANOVA followed by Dunnett’s post hoc test. ns, not significant (*p* > 0.05); **p < 0.01; ***p < 0.001; ****p < 0.0001

Pre-established (48 h) IXDRAb biofilms were significantly disrupted following treatment with sub-inhibitory concentrations (¼ MIC and ½ MIC) of *p*-CA (*p* < 0.05, Fig. 3). Exposure to ½ MIC achieved maximum eradication levels reaching 71.3%, whereas treatment with ¼ MIC resulted in a 45.8% reduction compared to untreated controls. Isolate-specific eradication profiles for all 32 IXDRAb isolates (Ac55–Ac100) are presented in Supplementary Figure S6. When isolates were stratified according to biofilm-forming capacity, both ¼ MIC and ½ MIC of *p*-CA significantly eradicated mature biofilms in strong and moderate biofilm formers (*p* < 0.05; Fig. 3B–C). In contrast, among weak biofilm formers, only treatment with ½ MIC resulted in a statistically significant reduction in pre-established biofilms (*p* < 0.05; Fig. 3D). Overall, higher eradication efficiency was consistently observed at ½ MIC across all biofilm phenotypes.

### The effect of p-coumaric acid on the metabolic activity of biofilm bacteria

The effect of ¼ MIC and ½ MIC *p*-CA on the metabolic activity of mature (48-h) biofilm-associated bacteria was assessed using the MTT assay. As presented in Supplementary Figure S7A, treatment with *p*-CA significantly decreased biofilm bacterial metabolic activity (*p* < 0.05), exhibiting metabolic activity reductions ranging from 21.90% to 39.5% at ¼ MIC and from 37.09% to 64.9% at ½ MIC. Isolate-specific responses are presented in Supplementary Figure S7B, demonstrating variability in the magnitude of metabolic inhibition among tested strains; however, ½ MIC *p*-CA consistently exerted greater metabolic suppression than ¼ MIC across all isolates.

### Mechanistic insights into antibiofilm activity of p-coumaric acid

#### Reduction of exopolysaccharides

To elucidate the mechanism underlying the eradication of pre-established IXDRAb biofilms, exopolysaccharide (EPS) production, a key structural component of the biofilm matrix, was quantified in strong biofilm-forming isolates (*n* = 12) using the phenol-sulfuric acid method. It was found that *p*-CA treatment significantly (*p* < 0.05) reduced EPS levels by 44.3% and 69% at ¼ MIC and ½ MIC, respectively, as displayed in Supplementary Figure S8.

#### Reduction in the hydrophobicity index

The MATH assay was employed to investigate whether the anti-biofilm activity of *p*-CA involves alterations in cell surface hydrophobicity. Results indicated a marked reduction in the hydrophobicity index of strong biofilm-forming-IXDRAb isolates (*n* = 12) treated with ¼ MIC and ½ MIC of *p*-CA, reaching 56% and 40.2%, respectively, compared to 76.3% in untreated controls (Supplementary Figure S9). These findings suggest that the reduction in surface hydrophobicity contributes, at least in part, to the observed biofilm inhibition.

#### Microscopy visualization of the antibiofilm effects of p-coumaric acid

**Fig. 4:**
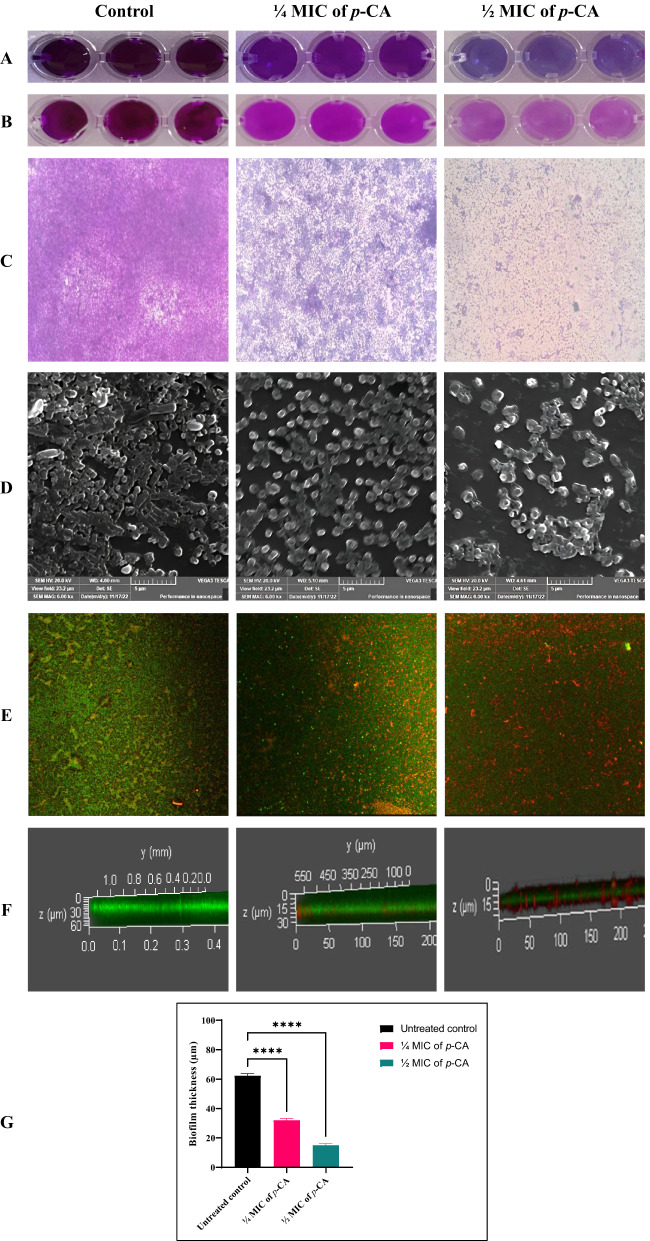
Multimodal evaluation of the therapeutic antibiofilm activity of sub-inhibitory p-coumaric acid (*p*-CA) against a representative strong biofilm-forming IXDR *Acinetobacter baumannii* isolate (Ac88). **A** Quantitative assessment of pre-established (48 h) biofilm eradication using the 96-well microtiter plate assay following treatment with sub-inhibitory concentrations of *p*-CA (¼ MIC and ½ MIC). **B** Effect of sub-inhibitory *p*-CA concentrations on the metabolic activity of biofilm-associated bacteria, as determined by the MTT assay. **C** Bright-field light microscopy images illustrating structural alterations in biofilms following treatment (400× magnification). **D** Scanning electron microscopy micrographs showing disruption of biofilm architecture and bacterial aggregation (scale bar = 5 μm). **E**–**F** Confocal laser scanning microscopy images of biofilms stained with acridine orange/propidium iodide (AO/PI), demonstrating biofilm viability and three-dimensional architecture in 2D and 3D, respectively. **G** Quantitative analysis of biofilm thickness derived from CLSM Z-stack measurements following treatment of 48 h pre-formed biofilms with sub-inhibitory *p*-CA concentrations. Data are presented as mean ± SD. Statistical significance was determined using one-way ANOVA; ****p < 0.0001 versus untreated control

To visually assess the impact of *p*-CA on mature IXDRAb biofilms, light microscopy (LM), scanning electron microscopy (SEM), and confocal laser scanning microscopy (CLSM) were performed on a 48-hour-old biofilm of a representative strong biofilm-forming isolate (**Ac88**). LM analysis at 400× magnification revealed a notable reduction in biomass in treated samples. SEM imaging demonstrated dense cell layers in the untreated control, while the ¼ MIC-treated biofilm appeared rough with clumped cells, and ½ MIC-treated samples exhibited sparse, individually dispersed cells. CLSM imaging using acridine orange (AO) and propidium iodide (PI) staining confirmed a decline in both viability and biofilm thickness post-treatment. Specifically, biofilm thickness was reduced by approximately 50% and 75% following treatment with ¼ MIC and ½ MIC of *p*-CA, respectively (Fig. 4).

#### Effect of sub-inhibitory concentration (½ MIC) of p-coumaric acid on expression of biofilm-related genes

**Fig. 5:**
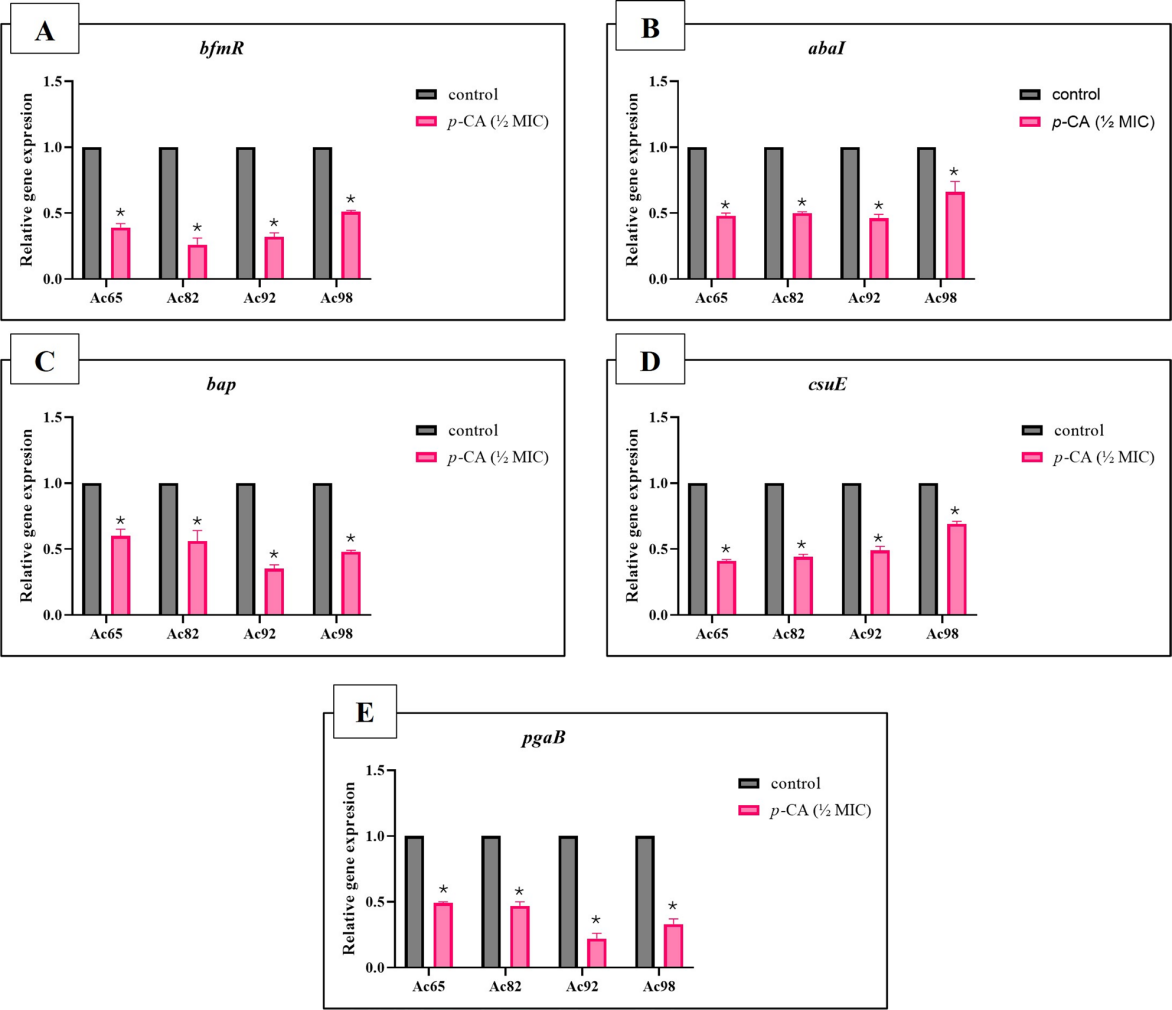
Relative expression levels of biofilm-associated genes in response to p-coumaric acid (*p*-CA) treatment at ½ MIC in strong biofilm-forming imipenem-resistant XDR *Acinetobacter baumannii* isolates (*n* = 4). Expression levels of (**A**) *bfmR*, (**B**) *abaI*, (**C**) *bap*, (**D**) *csuE*, and (**E**) *pgaB* were quantified by RT-qPCR and compared to untreated control samples. Gene expression levels were normalized to the housekeeping gene (*16 S rRNA*) and expressed as relative fold change using the 2^–ΔΔCT^ method. Bars represent mean fold change ± standard deviation (SD). Asterisks indicate statistically significant downregulation compared with untreated control for each isolate (*p < 0.05, unpaired t-test)

Quantitative real-time PCR (RT-qPCR) was conducted to evaluate the transcriptional alterations in five biofilm-associated genes (*bfmR*,* abaI*,* bap*,* csuE*, and *pgaB*) following exposure to *p*-CA at ½ MIC, compared to untreated controls. The fold-change analysis revealed that *p*-CA treatment resulted in a statistically significant downregulation (*p* < 0.05) of these genes across all four strong biofilm-producing IXDRAb isolates (Ac65, Ac82, Ac92, and Ac98), indicating its potential in impairing biofilm formation at the molecular level, as shown in Fig. 5. These findings indicate that *p*-CA can impair biofilm formation at the molecular level by suppressing the expression of critical biofilm determinants.

#### In silico molecular docking study on the target protein

The interaction between *p*-CA and the BfmR protein was investigated to better understand the likely mechanism by which it can produce its antibiofilm activity against *A. baumannii*. Molecular docking was carried out with the Glide ligand-docking module in standard precision mode. The findings demonstrated that *p*-CA was oriented correctly in the BfmR protein’s active region, with a substantial docking score of − 9.2 kcal mol⁻¹. As illustrated in Supplementary Figure S10, *p*-CA formed a complex with the BfmR protein by establishing a strong hydrogen bond with Ala106, while the aromatic ring of *p*-CA potentially engaged in π-stacking or π-hydrophobic interactions with nearby aromatic or hydrophobic residues Tyr104 and Hydrophobic contacts with Val105, Val109, and Val113. These in silico findings suggested that *p*-CA may interfere with BfmR function by binding to its regulatory or DNA-binding domains, potentially affecting its ability to activate transcription of downstream biofilm genes, providing preliminary structural insights into a possible interaction with BfmR that may contribute to the observed antibiofilm effects of *p*-CA against *A. baumannii.*

### In vivo efficacy and safety of p-coumaric acid–based therapy in an immunocompromised rat model

Cyclophosphamide administration resulted in profound multi-lineage myelosuppression in treated rats. This was evidenced by a marked reduction in mean total white blood cell (WBC) count by 88.6% (from 4,400 to 500 cells/mm³). Similarly, mean neutrophil, lymphocyte, and monocyte counts decreased by 65.4% (202.4 to 70 cells/mm³), 91.2% (3,916 to 345 cells/mm³), and 67.8% (264 to 85 cells/mm³), respectively. These findings confirmed the successful induction of an immunocompromised state, characterized by severe leukopenia, neutropenia, lymphopenia, and monocytopenia (Supplementary Figure S11). After the rats were infected with challenging *A. baumannii* (**Ac88**) intranasally, pharmacological therapy began 6 h after the infection with two other consecutive doses at 24 h and 48 h time points post-infection. The clinical symptoms, weight loss, and death rate were monitored for 7 days following the infection. The rats of groups 2 and 3 displayed more severe clinical scores than those of groups 4 and 5 (Supplementary Figure S12). Similarly, groups 2, 3, and 4 rats lost about 26.5, 29, and 11.4% of their initial body weight, respectively. In contrast, the rats in group 5 that received the combination rescue generally recovered to their pre-inoculation body weights by day 3, and the rats continued to gain weight until day 7.

**Fig. 6:**
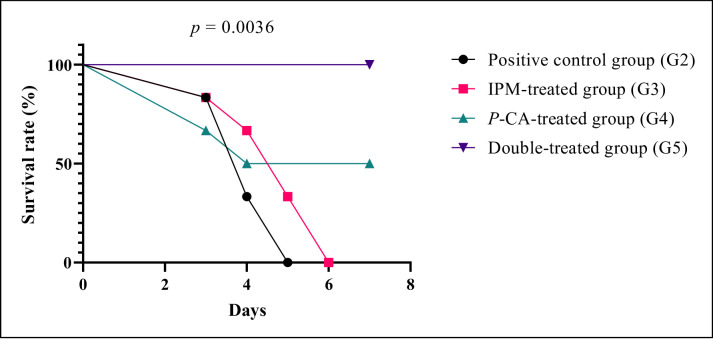
The effect of imipenem (IPM), p-coumaric acid (*p*-CA), and the double combination of imipenem and p-coumaric acid on rats’ survival. Statistical analysis by Log-rank (Mantel-Cox) test showed significant differences (*p* < 0.05) upon comparison of survival rates among treatment groups

A statistically significant disparity in median survival rates was observed between the combination therapy group and the remaining treatment groups (*p* < 0.05, Fig. 6). Notably, the double combination of IPM and *p*-CA ensured complete survival (100%) in the treated cohort over the 7-day observation period, aligning with in vitro data, supporting its in vivo efficacy in this immunocompromised rat pneumonia model and suggesting potential therapeutic usefulness that warrants further investigation. On the other hand, 100% of the rats in groups 2 and 3 died on days 5 and 6, respectively.

**Fig. 7:**
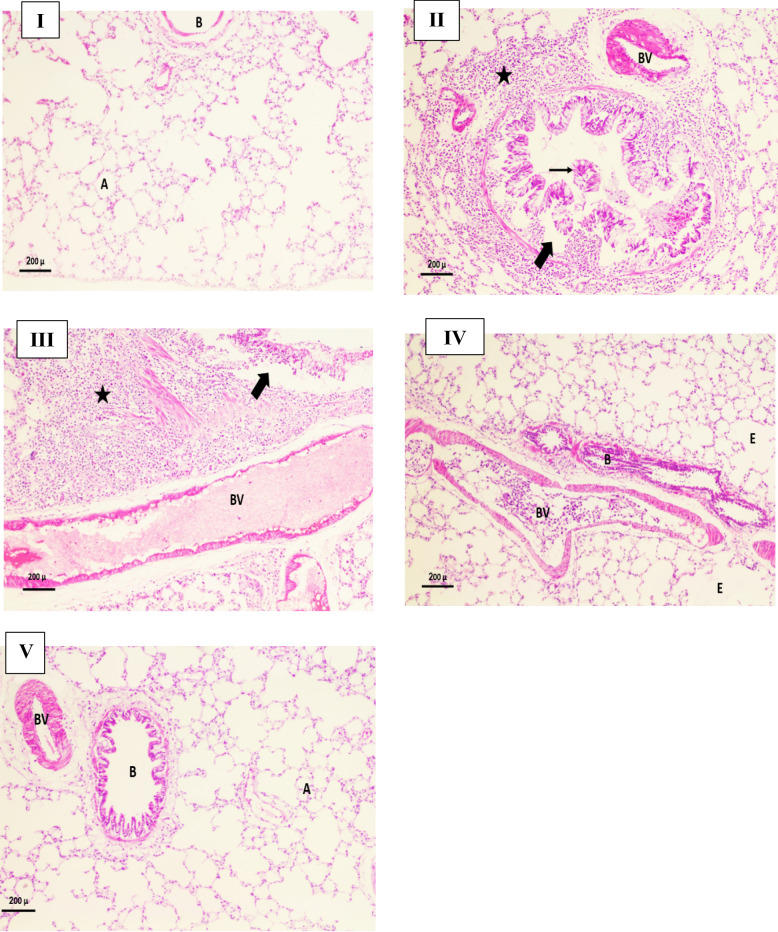
Representative hematoxylin and eosin (H&E)-stained pulmonary tissue sections from experimental rat groups (**I**) Negative control group demonstrating typical lung morphology with patent alveoli and intact parenchymal structure (A) and normal bronchiole (B). (**II**) Bacterium-infected positive-control group showing disruption of the wall of bronchiole with separation of its lining epithelium from the underlying smooth muscle (thick arrow), desquamated part of lining epithelium within the lumen (thin arrow), dilated blood vessel (BV) and marked inflammatory infiltration (*). (**III**) The imipenem-treated group showing failure of therapy represented in the separation of lining epithelium of bronchiole from the underlying smooth muscle (thick arrow), dilated congested blood vessel (BV), and marked inflammatory infiltration (*). (**IV**) The p-coumaric acid-treated group showing sparsely slight improvement represented by moderate disruption of histological architecture with areas of emphysema (E), irregularity of the wall of bronchiole (B) and markedly dilated congested blood vessel (BV). (**V**) The double-combination rescue group showing more normal histological architecture with normal alveolar spaces (A) and normal bronchiole (B) except for mildly dilated blood vessel (BV). Magnification, x100

Analysis of pulmonary tissue from the **Ac88**-infected positive control group exhibited disruption of the wall of the bronchiole with separation of its lining epithelium from the underlying smooth muscle, desquamated part of lining epithelium within the lumen, dilated blood vessel (BV) and marked inflammatory infiltration (Fig. 7II). Imipenem therapy group 2 and the single *p*-CA group showed absence and slight improvement, respectively (Fig. 7III and IV). Remarkably, the dual combination regimen effectively restored pulmonary function and histoarchitecture, as evidenced by the presence of well-aerated alveoli and normal lung tissue organization observed 72 h after treatment administration. (Fig. 7V).

**Fig. 8:**
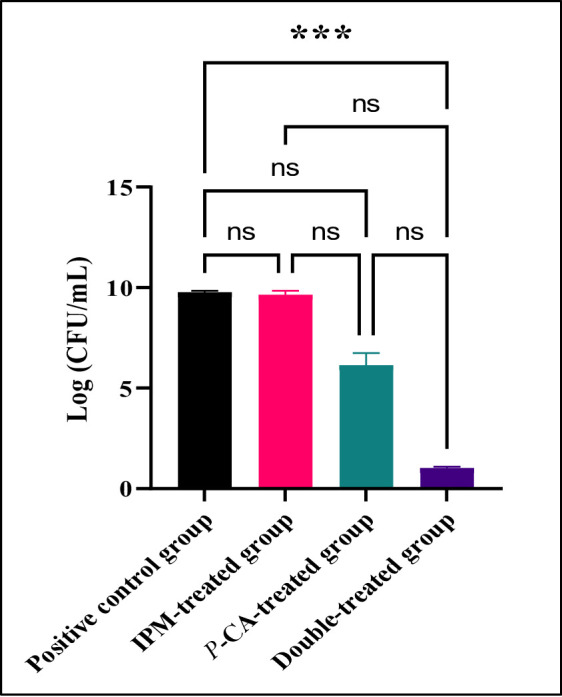
Quantitative bacteriological assessment of lung homogenates 72 h after *Acinetobacter baumannii* (**Ac88)** infection. Bacterial loads were enumerated by serial dilution plating and expressed as log_10_ CFU per gram lung tissue (mean ± SD, *n* = 3 biological replicates). ns; not significant, *p* > 0.05, ***Denotes statistically significant reduction (*p* < 0.001) versus control groups by one-way ANOVA with post-hoc Tukey test

The histopathological evaluation demonstrated abundant *A. baumannii* coccobacillary clusters in Giemsa-stained sections from infected controls. In contrast, these bacterial aggregates were completely absent in tissue samples obtained from the dual-therapy rescue group at the 72-hour post-treatment interval, coinciding with the observed clinical recovery (Supplementary Figure S13). The combination therapy group exhibited a profoundly significant decrease in viable bacterial counts relative to comparator groups (*p* < 0.001, Fig. 8).

Importantly, Histopathological examination revealed no evidence of extrapulmonary dissemination in *p*-CA–treated animals, as indicated by the absence of detectable pathological alterations in splenic and hepatic tissues (Supplementary Figure S14). In addition, no treatment-related histological abnormalities were observed, suggesting a lack of overt tissue toxicity at the tested doses. The cytotoxic potential of *p*-CA was further evaluated in vitro using normal human skin fibroblast (HSF) cells by the MTT assay. It was found that *p*-CA exhibited a half-maximal inhibitory concentration (IC₅₀) of 294.2 µg/mL (Supplementary Figure S15). Notably, all concentrations employed in the in vitro and in vivo experiments were substantially below this IC₅₀ value, indicating an acceptable safety margin under the experimental conditions.

## Discussion

The increasing prevalence of extensively drug-resistant *Acinetobacter baumannii* (XDRAb) continues to compromise the management of healthcare-associated infections, particularly in immunocompromised patients. In this study, 32% of clinical isolates recovered from oncology patients were imipenem-resistant XDR *A. baumannii* (IXDRAb), and all exhibited carbapenem resistance, underscoring the limited utility of broad spectrum of antibiotics, including cephalosporins, monobactams, aminoglycosides, fluoroquinolones, and carbapenems in this setting. Although colistin remained active in vitro, reliance on polymyxins is clinically constrained by nephrotoxicity, which is especially problematic in oncology populations receiving nephrotoxic regimens [36, 37].

Our findings demonstrate that p-coumaric acid (*p*-CA) has measurable antibacterial activity against IXDRAb and, importantly, acts as a promising adjuvant candidate that markedly restores imipenem activity. Subinhibitory *p*-CA substantially reduced imipenem MICs, with susceptibility restored in nearly all resistant isolates, indicating clinically relevant resistance modulation. While the present study was not designed to define a single mechanism, published evidence suggests that phenolic acids can enhance antibiotic activity by perturbing membrane permeability and enhancing antibiotics uptake, thereby increasing intracellular antibiotic exposure [9]. Beyond its activity, *p*-CA, a naturally occurring phenolic compound with a well-documented safety profile, demonstrated negligible cytotoxicity in vitro and is widely regarded as safe in dietary exposure [8].

Biofilm-associated persistence further amplifies the clinical challenge of IXDRAb, and all isolates in this study were biofilm formers. At subinhibitory concentrations, *p*-CA significantly inhibited biofilm formation and reduced established biofilm biomass, accompanied by decreased metabolic activity, reduced EPS production, and lower cell surface hydrophobicity. Consistent with these phenotypes, *p*-CA downregulated key biofilm-associated determinants (*bfmR*,* abaI*,* bap*,* csuE*,* pgaB*), supporting a multi-target antibiofilm effect. Docking analysis suggested favorable binding to BfmR, providing preliminary structural insight into a possible interaction that may contribute to transcriptional suppression, although biochemical validation is required.

The in vivo pneumonia model corroborated the in vitro observations, as the *p*-CA and imipenem combination improved survival, mitigated weight loss, reduced lung bacterial burden, and improved histopathological outcomes relative to monotherapy. Together, these data support *p*-CA as a promising antibiofilm adjuvant capable of potentiating carbapenems and reducing dependence on polymyxins for IXDRAb infections. Future work should prioritize pharmacokinetic and pharmacodynamic characterization, formulation optimization, and expanded in vivo testing in additional infection models to better define the translational potential of *p*-CA–based adjuvant strategies.

This study has several limitations. First, the analysis was based on clinical isolates obtained from a single center, which may limit the generalizability of the findings across diverse geographic regions. Second, although the immunocompromised rat pneumonia model provides valuable proof-of-concept evidence, animal models cannot fully capture the complexity of human XDRAb infections, including host immune variability and clinical comorbidities. Third, the mechanistic and docking analyses are exploratory; definitive confirmation of the molecular targets and pathways involved will require additional biochemical and genetic studies. Finally, comprehensive pharmacokinetic, pharmacodynamic, and long-term safety data for *p*-CA in humans are currently unavailable, and any potential clinical application will necessitate rigorous preclinical assessment.

## Conclusion

p-Coumaric acid potentiated imipenem activity and exerted significant antibiofilm effects against imipenem-resistant XDR *A. baumannii* from cancer patients, as evidenced by transcriptional suppression of biofilm determinants and improved outcomes in an immunocompromised rat pneumonia model. These findings locate *p*-CA as a multifunctional adjuvant candidate that targets both resistance and biofilm formation, warranting further clinical evaluation for managing XDR *A. baumannii* infections in vulnerable cancer populations.

## Supplementary Information

Below is the link to the electronic supplementary material.

Supplementary Material 1.

## Data Availability

No datasets were generated or analysed during the current study.
