# Supplementary material for: Unlocking p-coumaric acid’s potential against biofilm-forming extensively drug-resistant Acinetobacter baumannii: in vitro and in vivo study
Source: Ann Clin Microbiol Antimicrob. 2026 Apr 11;25:20. doi: 10.1186/s12941-026-00859-2 (PMC13123207; doi:10.1186/s12941-026-00859-2)
Supplement: Supplementary file 1 — Supplementary Material 1. [file 12941_2026_859_MOESM1_ESM.pdf]

Supplementary Material

Supplementary Methods

Supplementary Methods S1. Biofilm formation assay (microtiter plate crystal violet method)

The biofilm-forming capability of *Acinetobacter baumannii* isolates was determined via the microtiter plate method with crystal violet staining. Overnight tryptic soy broth (TSB; Oxoid, UK) cultures (0.5 McFarland) were inoculated into glucose-supplemented TSB and incubated statically (37 °C, 48 h). After washing with PBS, fixing and staining with crystal violet (0.1%, 15 min), the bound dye was solubilized in 33% glacial acetic acid. Isolates' optical density (OD_{595}) was measured [1]. Isolates were classified as non-, weak, moderate, or strong biofilm producers based on isolates' OD_{595} compared to the cut-off OD (ODc). The negative control's mean optical density (OD) plus three standard deviations (SD) was designated as the ODc. The extent of biofilm development was documented as follows: strong biofilm formation ($4 \times ODc < OD$), moderate biofilm formation ($2 \times ODc < OD \leq 4 \times ODc$), weak biofilm formation ($ODc < OD \leq 2 \times ODc$), and no biofilm formation ($OD \leq ODc$) [2].

Supplementary Methods S2. Antibiofilm activity of p-coumaric acid at subinhibitory concentrations

Preventive Antibiofilm Assay

To simulate prophylactic conditions, this assay evaluated the preventive efficacy of *p*-CA as a surface-coating or pre-exposure treatment to inhibit early biofilm formation when bacteria initially contact a surface in the presence of the compound, 100 µL of each IXDRAb isolate (adjusted to $OD_{560} = 0.02$, approximately 1.0×10^6 CFU/mL) in CAMHB supplemented with 1% glucose was inoculated into 96-well plates and incubated statically at 37 °C for 4 h. Then the plates were removed from the incubator and 100 µL of *p*-CA solution in media was added in a sequence in each well to give a final concentration equivalent to ¼ MIC & ½ MIC. The plates were subsequently subjected to static incubation at 37°C for an additional 24 h [3,4]. Biofilm biomass was quantified using the crystal violet staining technique [1] . All the experiments were conducted in triplicate. Wells without dissolved *p*-CA served as positive controls for biofilm formation. The biofilm biomass was quantified by measuring the OD at 595 nm. The Biofilm inhibition (%) was determined using the formula [5].

$$\% \, Biofilm\ inhibition = 100 \ - (\frac{OD_{595}\ treated}{OD_{595}\ positive\ control}) \times \ 100$$

Therapeutic Antibiofilm Assay

This assay was designed to evaluate the ability of *p*-CA to disrupt or eradicate pre-established, mature biofilms. To reflect the therapeutic challenge of treating existing biofilm-associated infections rather than preventing their initial formation, IXDRAb isolates were grown statically in 96-well flat base plates for 48 h to allow mature biofilm formation [1]. After discarding the planktonic cells, 200 µL of *p*-CA solution in CAMHB medium at subinhibitory concentrations of ¼ MIC and ½ MIC were sequentially added to each well and cultured for an additional 24 h. Each experiment was conducted in triplicate, with wells containing CAMHB and the solvent 0.5% DMSO, devoid of dissolved *p*-CA, serving as the positive controls for biofilm formation. The protocol mentioned above was followed to quantify the antibiofilm potential [5].

Supplementary Methods S3. MTT assay for metabolic activity of biofilm cells

The MTT (3-(4,5-dimethylthiazol-2-yl)-2,5-diphenyltetrazolium bromide) assay was used to assess the metabolic activity of biofilm-embedded bacteria treated with p-coumaric acid (*p*-CA) based on reduction of MTT to formazan by viable cells [6], [7]. The same protocol of mature biofilm formation and *p*-CA treatment was previously described [1], [5]. Then, biofilms were incubated with MTT (0.05%) for 3 h; formazan crystals were solubilized in DMSO and measured at OD_{520}. The percentage reduction in metabolic activity was as follows:

$$\% \, Metabolic\ activity\ reduction = 100 \ - (\frac{OD_{520}\ treated}{OD_{520}\ positive\ control}) \times \ 100$$

Supplementary Methods S4. Exopolysaccharides (EPS) quantification (phenol–sulfuric acid assay)

Mature biofilms of tested strong biofilm producers were incubated with/without *p*-CA for 24 h. EPS was precipitated using cold ethanol and quantified by the phenol–sulfuric acid method as previously established [8]. The percentage reduction of EPS following *p*-CA treatment was [9] :

$$\% \, EPS\ reduction = 100 - (\frac{OD_{490}\ treated}{OD_{490}\ control}) \times \ 100$$

Supplementary Methods S5. Cell surface hydrophobicity by Microbial adhesion to hydrocarbons (MATH) assay

Cell surface hydrophobicity (CSH) was assessed for each strong biofilm-forming treated with and without *p*-CA using MATH assay. This method evaluates bacterial affinity for the hydrophobic hydrocarbon toluene, following the previously described protocol [10,11]. The hydrophobicity index (%) was:

$$\%\, Hydrophobicity\ index\ = 100\ - (\frac{Final\ OD_{530}\ after\ vortex}{Initial\ OD_{530}\ before\ vortex}) \times\ 100$$

Supplementary Methods S6. Microscopy analysis of antibiofilm activity of p-coumaric acid

A representative isolate (**Ac88**) was selected to visualize the impact of *p*-CA on mature biofilms using light microscopy, scanning electron microscopy, and confocal laser scanning microscopy.

Light microscopic visualization of p-coumaric acid–induced biofilm disruption

Mature biofilms on glass coverslips were treated with *p*-CA (¼ or ½ MIC, 24 h), then stained with crystal violet and examined at 400× magnification, as illustrated previously [12,13].

Ultrastructural analysis by scanning electron microscopy

Biofilms were fixed with 2.5% glutaraldehyde (30 min, 37 °C), dehydrated through ethanol series, sputter-coated with gold, and analyzed using a Hitachi S-34002N SEM, following a previously described protocol [2].

Three-dimensional imaging of biofilm using confocal laser scanning microscopy

Preformed biofilms were stained with acridine orange (live cells, green) and propidium iodide (dead cells, red), then imaged using a Leica DMi8 CLSM, as previously detailed [14].

Supplementary Methods S7. Quantitative real-time PCR analysis workflow

Total RNA was extracted from four strong biofilm-producing isolates cultured in TSB with or without *p*-CA (½ MIC, 24 h, 37 °C) using the PureLink™ RNA Mini Kit, following the manufacturer's protocol. cDNA was synthesized using the First Strand cDNA Synthesis Kit. RT-qPCR was performed with Power SYBR™ Green Master Mix and gene-specific primers, as previously described [15–17]. In both *p*-CA-treated and untreated samples, *16S rRNA* was used as a housekeeping gene [18]. The primer sequences are demonstrated in **Table 1**. The relative

expression levels of the target genes were quantified using the $2^{-\Delta\Delta CT}$ method, as described by [19], which is widely used for relative quantification in RT-qPCR analyses. The threshold cycle (CT) represents the cycle at which fluorescence exceeds a predetermined threshold, indicating detectable levels of amplified product. Gene expression was normalized to that of the housekeeping gene *16S rRNA*. The calculations were performed using the following equations:

- ΔCT (treated) = CT (target gene, treated) – CT (reference gene, treated)
- ΔCT (control) = CT (target gene, control) – CT (reference gene, control)
- ΔΔCT = ΔCT (treated) – ΔCT (control)
- Relative expression (fold change) = $2^{-\Delta\Delta CT}$

Table 1: Primer sequences used for evaluating gene expression in RT-qPCR analysis

Gene	Sequence	Reference
abaI	F 5′-CCC GCA GCA CGT AAT AAA CG-3′<br>R 5′-AGC AGT CAG GCT GTG TCA TC-3′	[15]
bfmR	F 5′-ATT CGT GCT TTG TTAC GCC G-3′<br>R 5′-GCG ATA AAA TAC GGC CAG CG-3′	[15]
bap	F 5′-TGC TGA CAG TGA CGT AGA ACC ACA-3′<br>R 5′-TGC AAC TAG TGG AAT AGC AGC CCA-3′	[16]
csuE	F 5′-CAT CTT CTA TTT CGG TCC C-3′<br>R 5′-CGG TCT GAG CAT TGG TAA-3′	[16]
pgaB	F 5′-AAG AAA ATG CCT GTG CCG ACC A-3′<br>R 5′-GCG AGA CCT GCA AAG GGC TGA T-3′	[17]
16S rRNA	F 5′-TGG CTC AGA TTG AAC GCT GGC GGC-3′<br>R 5′-TAC CTT GTT ACG ACT TCA CCC CA-3′	[18]

Supplementary Methods S8. *In vitro* cytotoxicity (MTT) assay

The cytotoxic potential of *p*-CA on the human skin fibroblast (HSF) normal cell line (National Research Centre, Cairo, Egypt) was assessed using the MTT assay, as previously described [20]. HSF cells were cultured in Dulbecco's Modified Eagle Medium (DMEM) supplemented with 10% foetal bovine serum (FBS), 100 U/mL penicillin, and 100 µg/mL streptomycin, and maintained at 37°C in a humidified atmosphere containing 5% CO_2. Serial two-fold dilutions of *p*-CA were prepared to test a range of concentrations. Confluent cell monolayers were seeded in 96-well microtiter plates and incubated for 24 h. The cells were then treated with different concentrations of *p*-CA in triplicate and incubated for 48 h under standard conditions.

After treatment, 20 μL of MTT solution (5 mg/mL) was added to each well and incubated for 4 h at 37°C. Following incubation, the medium was gently aspirated, and 150 μL of DMSO was added to dissolve the formazan crystals. The plates were covered with aluminum foil and placed on an orbital shaker for 15 minutes. The absorbance was measured at 570 nm using a microplate reader (B.M.G. Labtech, FLUOstar Omega, Ortenberg, Germany). The half-maximal inhibitory concentration (IC_{50}), indicating the concentration of *p*-CA required to inhibit 50% of cell viability, was calculated using dose-response curve.

Supplementary Tables

Table S1: Antibiotic resistance profiles of 32 clinical imipenem-resistant XDR *Acinetobacter baumannii* isolates and their biofilm formation capacity

Source	Isolate code	BF	A	B		C				D			E			F		G			H	I	MAR
			PRL	TZP	SAM	CAZ	FEP	CTX	CRO	IPM	MRP	DRP	CN	TOB	AK	TE	DO	CIP	LEV	GAT	SXT	CT	
t. aspirate	**Ac55**	**M**	R	R	R	R	R	R	R	R	R	R	S	S	R	S	S	R	I	I	R	S	**0.65**
t. aspirate	**Ac58**	**St**	R	R	R	R	R	R	R	R	I	I	I	I	R	S	S	R	R	R	R	S	**0.65**
t. aspirate	**Ac64**	**M**	R	R	R	R	R	R	R	R	R	R	S	S	R	I	I	R	R	S	R	S	**0.7**
t. aspirate	**Ac65**	**St**	R	R	R	R	R	R	R	R	R	R	S	S	R	S	S	R	R	I	R	S	**0.7**
t. aspirate	**Ac66**	**M**	R	R	R	R	R	R	R	R	R	R	S	S	R	S	S	R	R	S	R	S	**0.7**
wound	**Ac67**	**St**	R	R	R	R	R	R	R	R	R	R	I	I	R	I	I	R	R	R	R	S	**0.75**
t. aspirate	**Ac68**	**W**	R	R	R	R	R	R	R	R	R	R	S	S	R	S	S	R	R	R	R	S	**0.75**
t. aspirate	**Ac69**	**M**	R	R	R	R	R	R	R	R	R	R	S	S	R	S	S	R	R	R	R	S	**0.75**
wound	**Ac70**	**M**	R	R	R	R	R	R	R	R	R	R	S	S	R	S	S	R	R	R	R	S	**0.75**
Bl	**Ac71**	**M**	R	R	R	R	R	R	R	R	R	R	S	S	R	S	S	R	R	R	R	S	**0.75**
wound	**Ac72**	**W**	R	R	R	R	R	R	R	R	R	R	S	S	R	S	S	R	R	R	R	S	**0.75**
t. aspirate	**Ac73**	**St**	R	R	R	R	R	R	R	R	R	R	S	S	R	S	S	R	R	R	R	S	**0.75**
wound	**Ac78**	**St**	R	R	R	R	R	R	R	R	R	R	R	R	R	S	S	R	R	I	R	S	**0.8**
wound	**Ac82**	**St**	R	R	R	R	R	R	R	R	R	R	R	R	R	S	S	R	R	R	R	S	**0.85**
t. aspirate	**Ac83**	**St**	R	R	R	R	R	R	R	R	R	R	R	R	R	S	S	R	R	R	R	S	**0.85**
Bl	**Ac84**	**M**	R	R	R	R	R	R	R	R	R	R	R	R	R	R	I	R	R	R	S	S	**0.85**
Bl	**Ac85**	**W**	R	R	R	R	R	R	R	R	R	R	R	R	R	R	I	R	R	R	I	S	**0.85**
Bl	**Ac86**	**M**	R	R	R	R	R	R	R	R	R	R	I	I	R	R	R	R	R	R	R	S	**0.85**
sputum	**Ac87**	**W**	R	R	R	R	R	R	R	R	R	R	R	R	R	R	R	R	R	R	I	S	**0.9**
t. aspirate	**Ac88**	**St**	R	R	R	R	R	R	R	R	R	R	R	R	R	R	R	R	R	R	I	S	**0.9**
t. aspirate	**Ac89**	**St**	R	R	R	R	R	R	R	R	R	R	R	R	R	R	R	R	R	R	I	S	**0.9**
wound	**Ac90**	**M**	R	R	R	R	R	R	R	R	R	R	R	R	R	R	R	R	R	R	I	S	**0.9**
sputum	**Ac91**	**W**	R	R	R	R	R	R	R	R	R	R	R	R	R	R	R	R	R	R	S	S	**0.9**
t. aspirate	**Ac92**	**St**	R	R	R	R	R	R	R	R	R	R	R	R	R	R	R	R	R	R	S	S	**0.9**
t. aspirate	**Ac93**	**M**	R	R	R	R	R	R	R	R	R	R	R	R	R	R	R	R	R	I	R	S	**0.9**
t. aspirate	**Ac94**	**M**	R	R	R	R	R	R	R	R	R	R	R	R	I	R	R	R	R	R	R	S	**0.9**
Bl	**Ac95**	**M**	R	R	R	R	R	R	R	R	R	R	R	R	R	R	I	R	R	R	R	S	**0.9**
t. aspirate	**Ac96**	**St**	R	R	R	R	R	R	R	R	R	R	R	R	R	R	R	R	R	R	R	S	**0.95**
Bl	**Ac97**	**M**	R	R	R	R	R	R	R	R	R	R	R	R	R	R	R	R	R	R	R	S	**0.95**
t. aspirate	**Ac98**	**St**	R	R	R	R	R	R	R	R	R	R	R	R	R	R	R	R	R	R	R	S	**0.95**
t. aspirate	**Ac99**	**M**	R	R	R	R	R	R	R	R	R	R	R	R	R	R	R	R	R	R	R	S	**0.95**
t. aspirate	**Ac100**	**M**	R	R	R	R	R	R	R	R	R	R	R	R	R	R	R	R	R	R	R	S	**0.95**

W, weak; M, moderate; St, strong. **Susceptibility interpretations**: R, resistant; I, intermediate; S, sensitive. Antibiotics tested (disc content): PRL (piperacillin, 100 µg), TZP (piperacillin-tazobactam, 100/10 µg), SAM (ampicillin-sulbactam, 10/10 µg), CAZ (ceftazidime, 30 µg), FEP (cefepime, 30 µg), CTX (cefotaxime, 30 µg), CRO (ceftriaxone, 30 µg), IPM (imipenem, 10 µg), MRP (meropenem, 10 µg), DRP (doripenem, 10 µg), CN (gentamicin, 10 µg), TOB (tobramycin, 10 µg), AK (amikacin, 30 µg), TE (tetracycline, 30 µg), DO (doxycycline, 30 µg), CIP (ciprofloxacin, 5 µg), LEV (levofloxacin, 5 µg), GAT (gatifloxacin, 5 µg), SXT (trimethoprim/sulfamethoxazole, 1.25/23.75 µg), CT (colistin).

Antibiotic categories

A: Penicillins, **B**: β-lactam combination agents, **C**: Cephems, **D:** Carbapenems, **E:** Aminoglycosides, **F**: Tetracyclines, **G:** Fluoroquinolones, **H:** Folate pathway antagonists, **I**: Lipopeptides.

MAR= Multiple Antibiotic Resistance index

Table S2: Modulatory effect of p-coumaric acid on imipenem resistance among XDR *Acinetobacter baumannii* isolates (*n* =32)

Isolate Code	IPM MIC (µg/mL)	IPM + ¼ MIC *p*-CA (µg/mL)	MF_1	IPM + ½ MIC *p*-CA (µg/mL)	MF_2
**Ac55**	64	8	8	1	64
**Ac58**	512	64	8	2	256
**Ac64**	256	32	8	1	256
**Ac65**	256	32	8	1	256
**Ac66**	128	16	8	2	64
**Ac67**	32	8	4	0.5	64
**Ac68**	128	64	2	2	64
**Ac69**	32	4	8	1	32
**Ac70**	128	64	2	1	128
**Ac71**	256	64	4	2	128
**Ac72**	64	32	2	1	64
**Ac73**	64	8	8	0.5	128
**Ac78**	32	4	8	0.5	64
**Ac82**	512	64	8	2	256
**Ac83**	128	16	8	0.5	256
**Ac84**	128	16	8	1	128
**Ac85**	32	8	4	1	32
**Ac86**	512	128	4	2	256
**Ac87**	256	128	2	4	64
**Ac88**	128	16	8	1	128
**Ac89**	128	32	4	1	128
**Ac90**	128	32	4	0.5	256
**Ac91**	128	32	4	2	64
**Ac92**	128	32	4	1	128
**Ac93**	64	8	8	0.5	128
**Ac94**	64	8	8	1	64
**Ac95**	32	8	4	0.5	64
**Ac96**	512	64	8	1	512
**Ac97**	64	8	8	1	64
**Ac98**	256	64	4	1	256
**Ac99**	32	4	8	0.5	64
**Ac100**	512	128	4	1	512

IPM (imipenem), *p*-CA (p-coumaric acid), MIC (minimum inhibitory concentration), MF (modulation factor = MIC of IPM alone ÷ MIC of IPM in combination)

Supplementary Figures

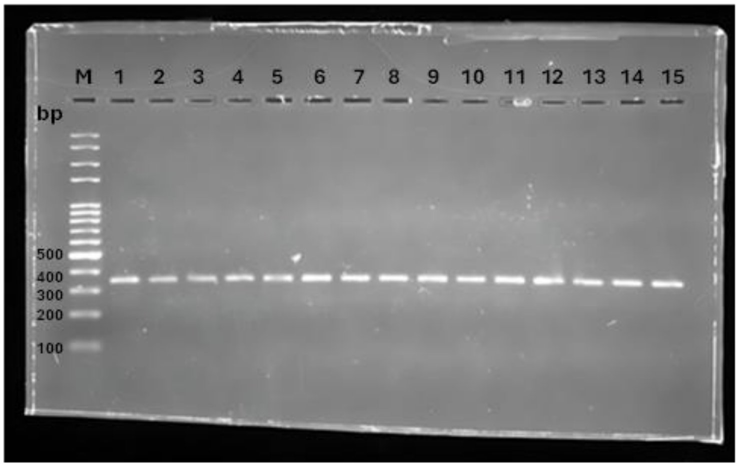

Figure S1: A representative electropherogram of PCR amplified products of *bla*$_{OXA\text{-}51\text{-}like}$ gene extracted from suspected *Acinetobacter baumannii* isolates. The lane, M; 100 bp DNA Ladder. Lanes 1-15 were amplified products of isolates at 353 bp

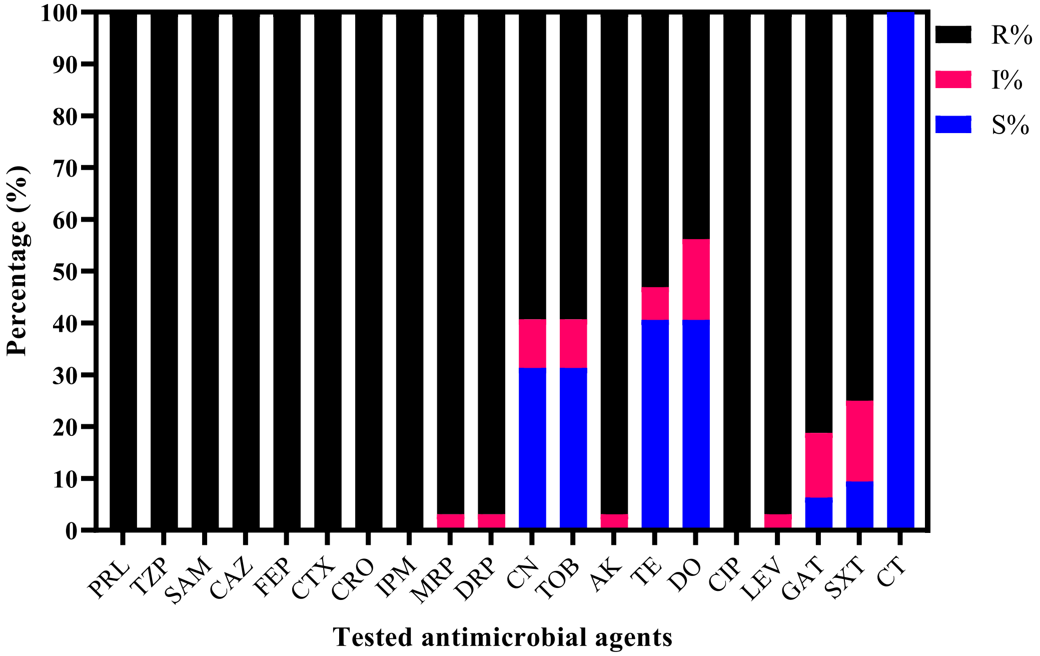

Figure S2: Incidence of resistance of the tested 32 isolates of *Acinetobacter baumannii* to different antimicrobials. R, I, and S refer to resistant, intermediate, and sensitive to antibiotics, respectively. piperacillin (PRL, 100 µg), piperacillin-tazobactam (TZP, 100/10 µg), ampicillin-sulbactam (SAM, 10/10 µg), ceftazidime (CAZ, 30 µg), cefepime (FEP, 30 µg), cefotaxime (CTX, 30 µg), ceftriaxone (CRO, 30 µg), imipenem (IPM, 10 µg), meropenem (MRP, 10 µg), doripenem (DRP, 10 µg), gentamicin (CN, 10 µg), tobramycin (TOB, 10 µg), amikacin (AK, 30 µg), tetracycline (TE, 30 µg), doxycycline (DO, 30 µg), ciprofloxacin (CIP, 5 µg), levofloxacin (LEV, 5 µg), gatifloxacin (GAT, 5 µg), trimethoprim/sulfamethoxazole (SXT, 1.25/23.75 µg), colistin (CT)

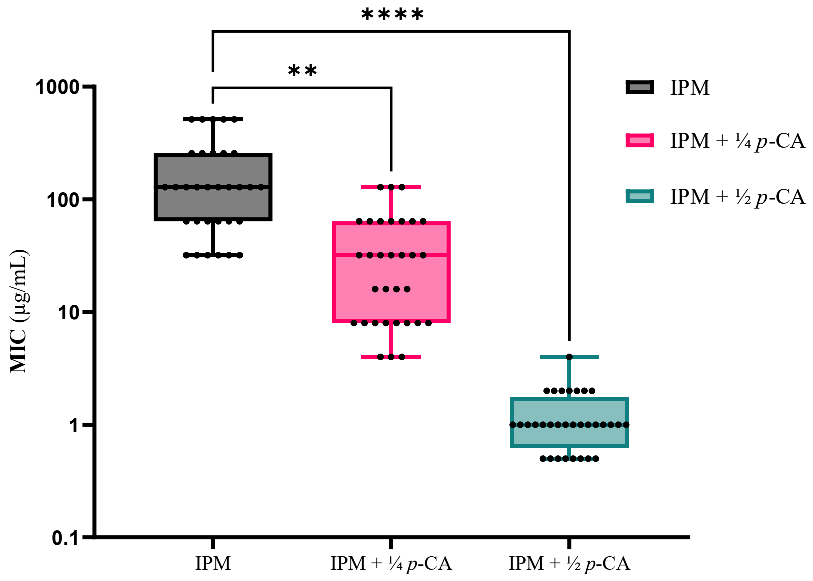

Figure S3: Box plots showing MICs of imipenem of the selected 32 imipenem-resistant XDR *Acinetobacter baumannii* clinical isolates in the presence/absence of sub-inhibitory p-coumaric acid (*p*-CA) concentrations (¼ MIC and ½ MIC). Box-and-whisker plots display the distribution of MICs of imipenem across the tested 32 IXDRAb isolates either untreated or treated with ¼ MIC or ½ MIC of *p*-CA compared to untreated controls. Box boundaries represent the 25th and 75th percentiles (interquartile range), horizontal lines within boxes indicate median values, and whiskers extend to minimum and maximum observations. Individual data points (black dots) are overlaid to illustrate the actual distribution of MICs of imipenem among the tested isolates. Statistical analysis was performed using one-way ANOVA followed by Dunnett's multiple comparison test, $^{**}p < 0.01$, $^{****}p < 0.0001$ vs. untreated control.

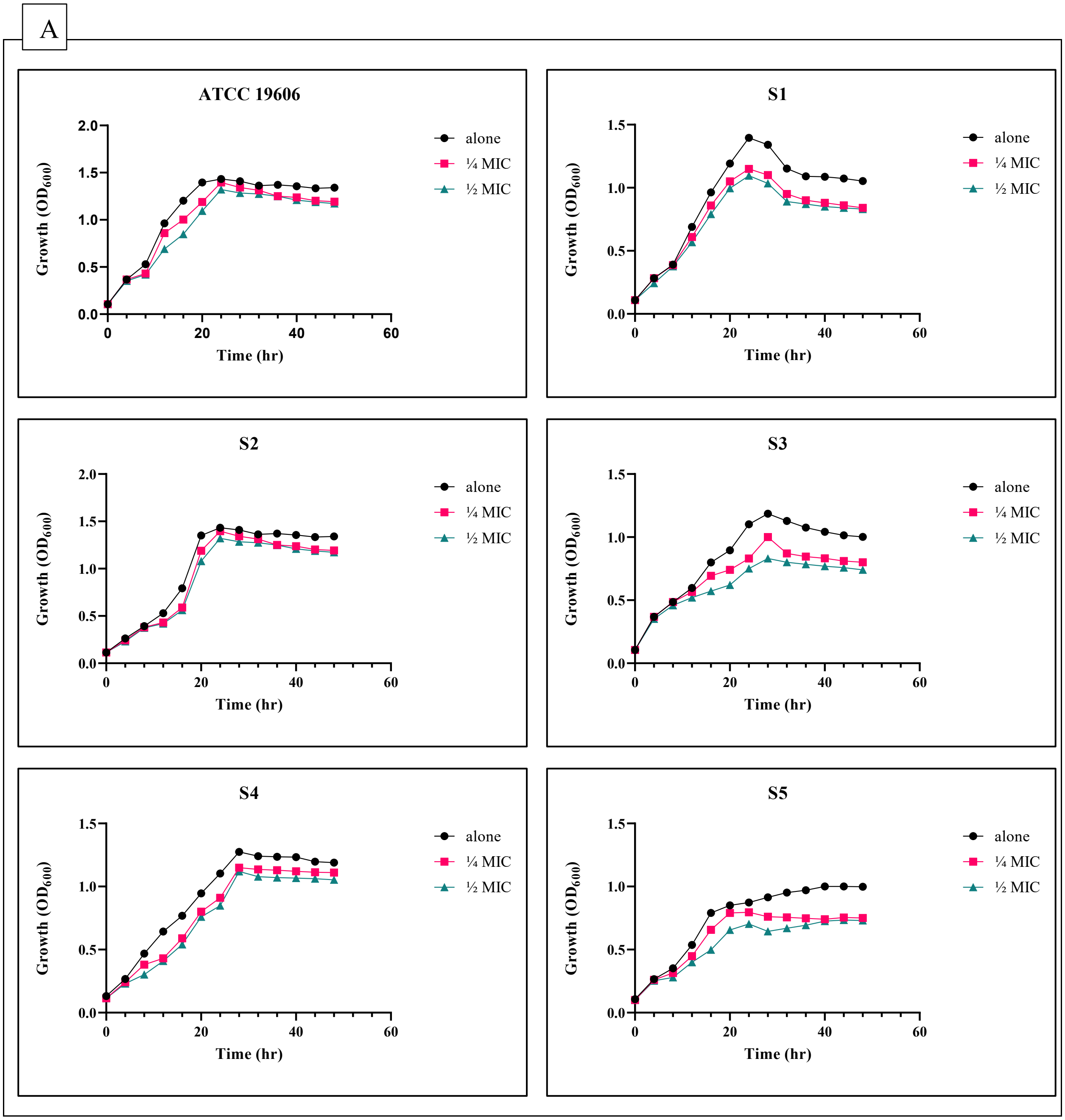
A
ATCC 19606
S1
S2
S3
S4
S5
Growth (OD600)
Time (hr)
alone
¼ MIC
½ MIC

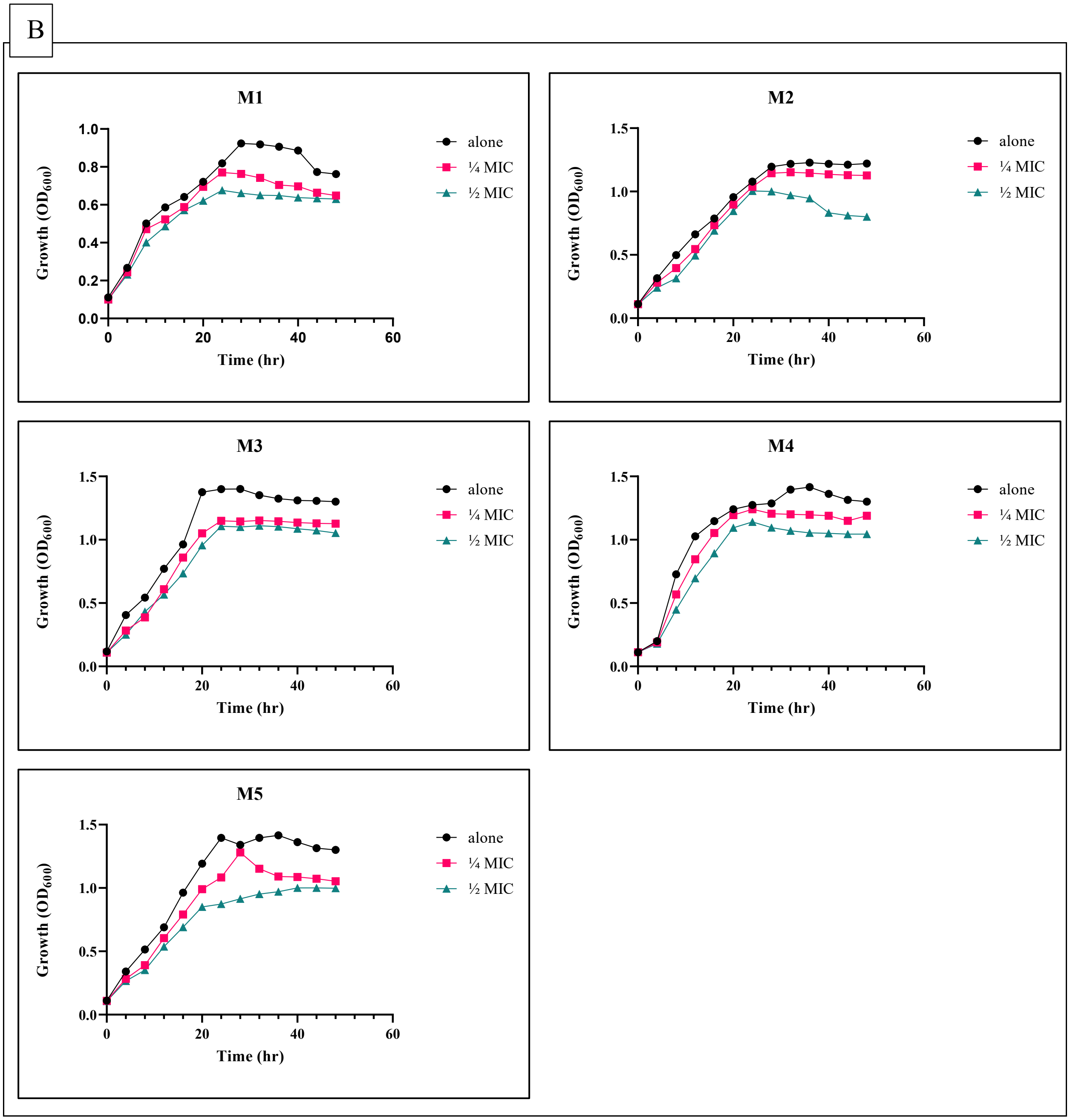
B
M1
M2
M3
M4
M5
Growth (OD600)
Time (hr)
alone
¼ MIC
½ MIC
0.0
0.2
0.4
0.6
0.8
1.0
0.5
1.5
0
20
40
60

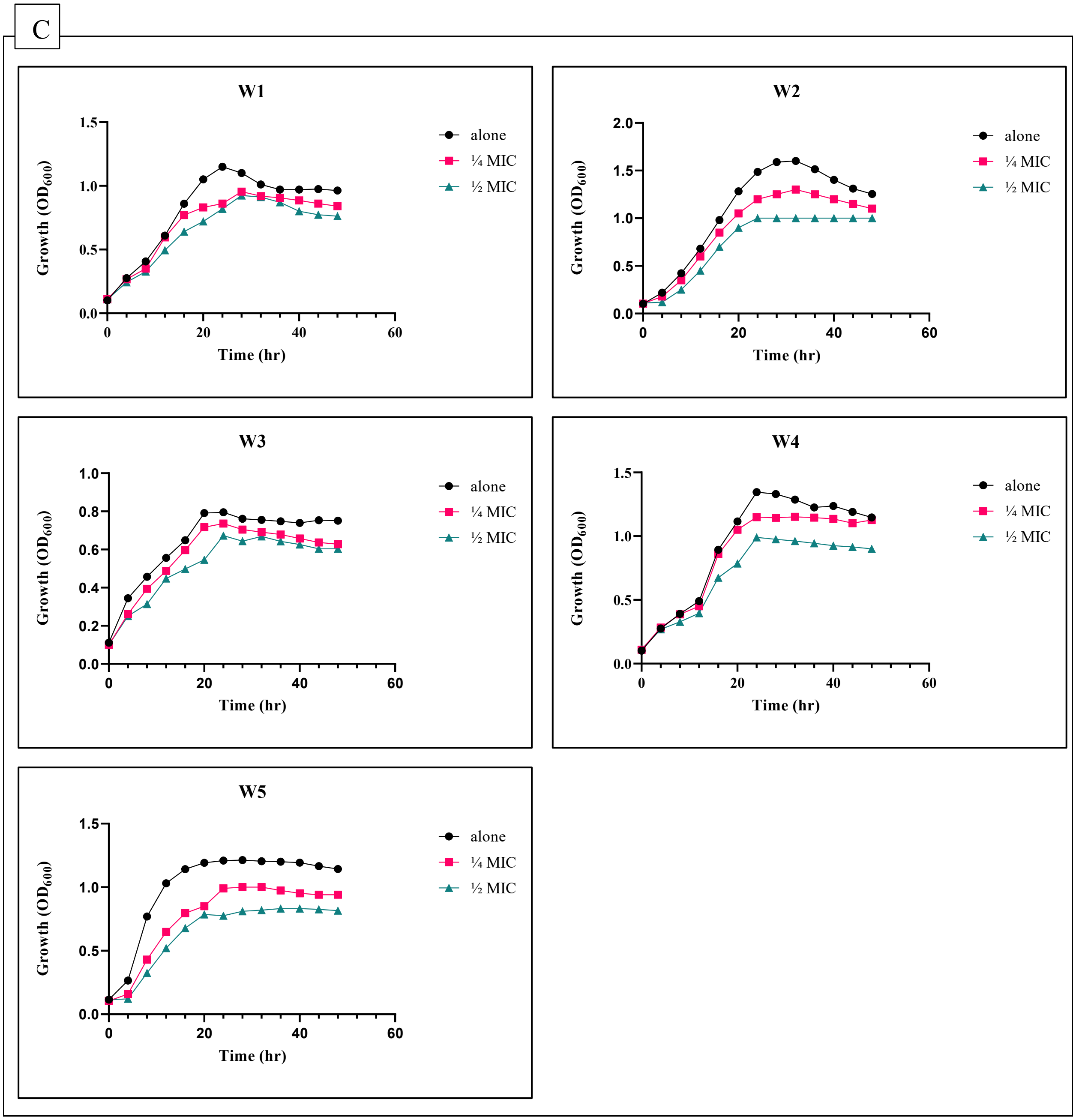

Figure S4: **(A-C)** Bacterial growth curves of selected *Acinetobacter baumannii* isolates in the presence of sub-inhibitory concentrations (¼ MICs and ½ MICs) of p-coumaric acid, (S) Strong biofilm formers, (M) Moderate biofilm formers, (W) Weak biofilm formers, each compared with their corresponding untreated controls. Bacterial growth was monitored by measuring optical density at 600 nm (OD_{600}) over 48 hours. Data represent mean values from three independent experiments. No statistically significant differences were observed between treated and untreated groups ($p > 0.05$, one-way ANOVA with Dunnett's post-hoc test)

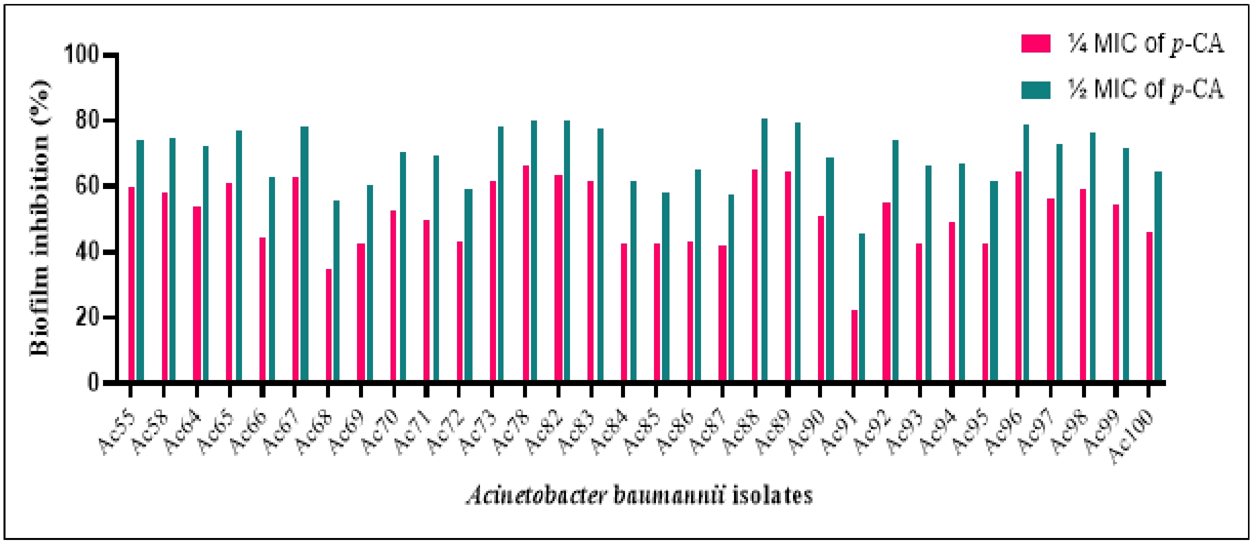

Figure S5: Preventive antibiofilm activity of sub-inhibitory p-coumaric acid (*p*-CA) concentrations against *Acinetobacter baumannii* clinical isolates (*n* = 32). Bars represent mean biofilm inhibition percentages for each isolate (Ac55–Ac100) after treatment with ¼ MIC or ½ MIC of *p*-CA.

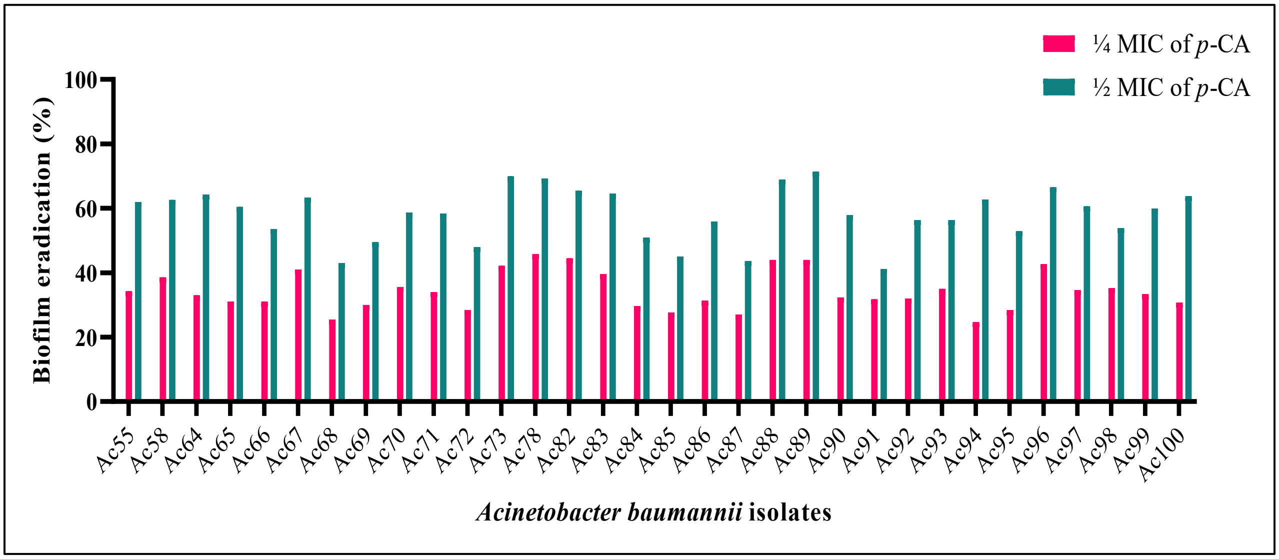

Figure S6: Therapeutic antibiofilm activity of sub-inhibitory p-coumaric acid (*p*-CA) concentrations against *Acinetobacter baumannii* clinical isolates (*n* = 32). Bars represent mean preformed biofilm eradication percentages for each isolate (Ac55–Ac100) after treatment with ¼ MIC or ½ MIC of *p*-CA.

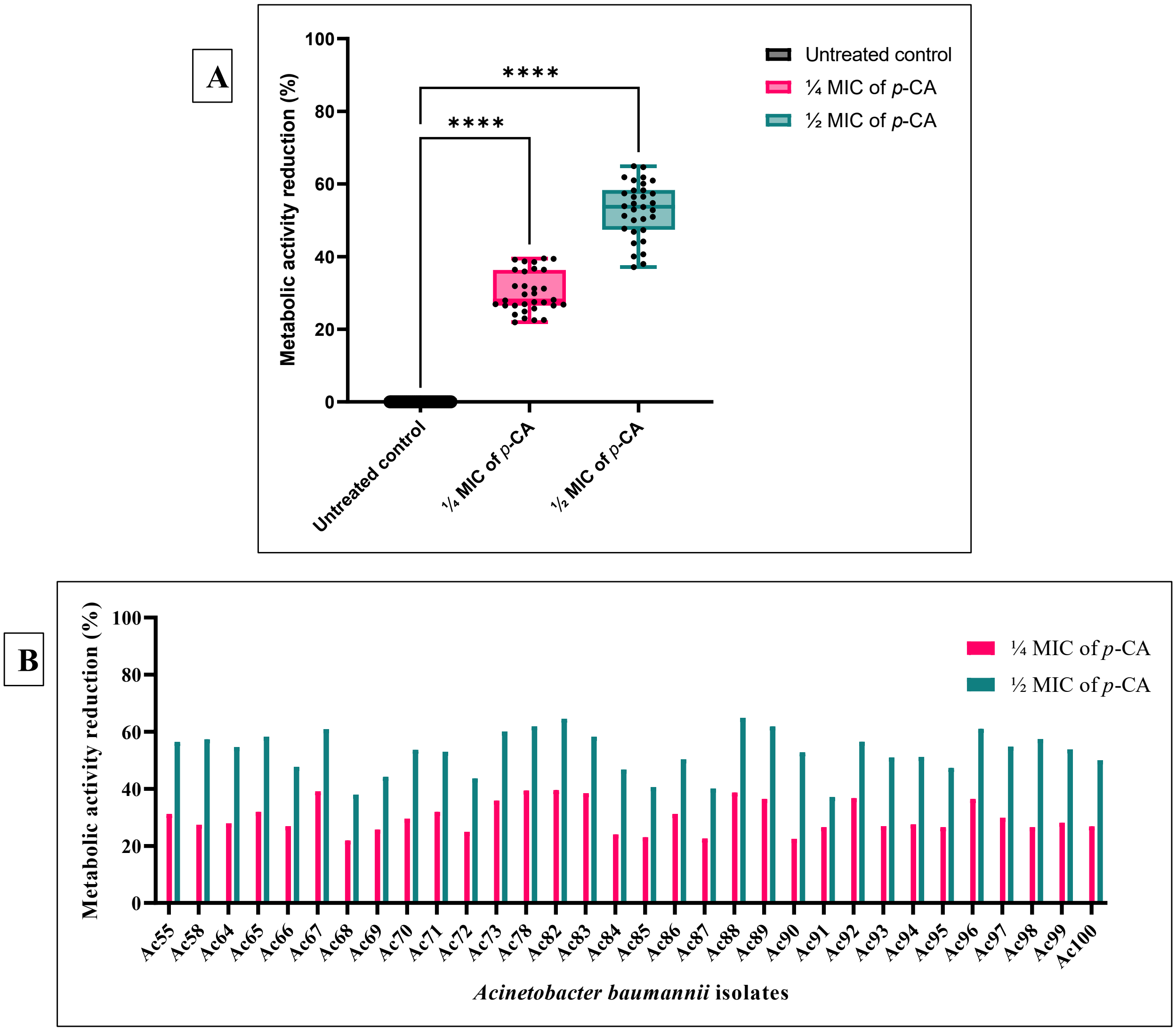

Figure S7: Effect of sub-inhibitory p-coumaric acid (*p*-CA) concentrations (¼ MIC and ½ MIC) on metabolic activity of biofilm-associated bacteria of imipenem-resistant XDR *Acinetobacter baumannii* clinical isolates ($n = 32$). Metabolic activity is expressed as a percentage of reduction relative to untreated control biofilms. **(A)** Box-and-whisker plots display the distribution of metabolic activity reduction percentages across the tested 32 IXDRAb isolates treated with ¼ MIC or ½ MIC of *p*-CA compared to untreated controls. Box boundaries represent the 25th and 75th percentiles (interquartile range), horizontal lines within boxes indicate median values, and whiskers extend to minimum and maximum observations. Individual data points (black dots) are overlaid to illustrate the actual distribution of metabolic activity reduction percentages among the tested isolates. Statistical analysis was performed using one-way ANOVA followed by Dunnett's multiple comparison test. ****$p < 0.0001$ vs. untreated control. **(B)** Bars represent mean metabolic activity reduction percentages for each isolate (Ac55–Ac100) after treatment with ¼ MIC or ½ MIC of *p*-CA.

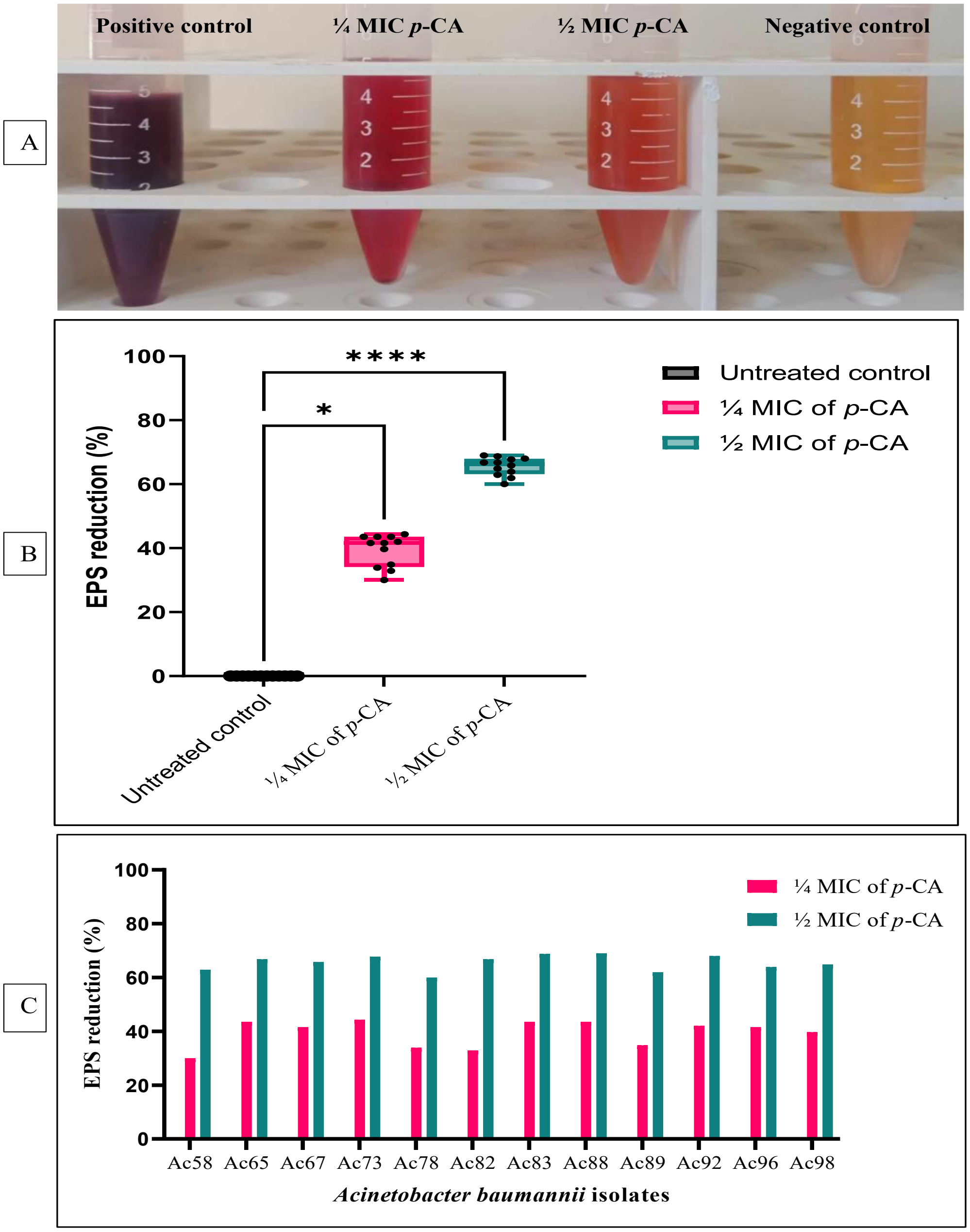

Figure S8: Effect of ¼ MIC and ½ MIC of p-coumaric acid (*p*-CA) on exopolysaccharides (EPS) production by strong biofilm-forming IXDRAb isolates (*n* = 12), assessed via the phenol-sulfuric acid method. **(A)** Representative images showing differences in red color intensity. **(B)** Box-and-whisker plots display the distribution of EPS reduction percentages across the tested isolates treated with ¼ MIC or ½ MIC of *p*-CA compared to untreated controls. Box boundaries represent the 25th and 75th percentiles (interquartile range), horizontal lines within boxes indicate median values, and whiskers extend to minimum and maximum observations. Individual data points (black dots) are overlaid to illustrate the actual distribution of EPS reduction percentages among the tested isolates. Statistical analysis was performed using one-way ANOVA followed by Dunnett's multiple comparison test $^{*}p < 0.05$; $^{****}p < 0.0001$ vs. untreated control. **(C)** Bars represent mean EPS reduction percentages for each strong biofilm-forming isolate after treatment with ¼ MIC or ½ MIC of *p*-CA.

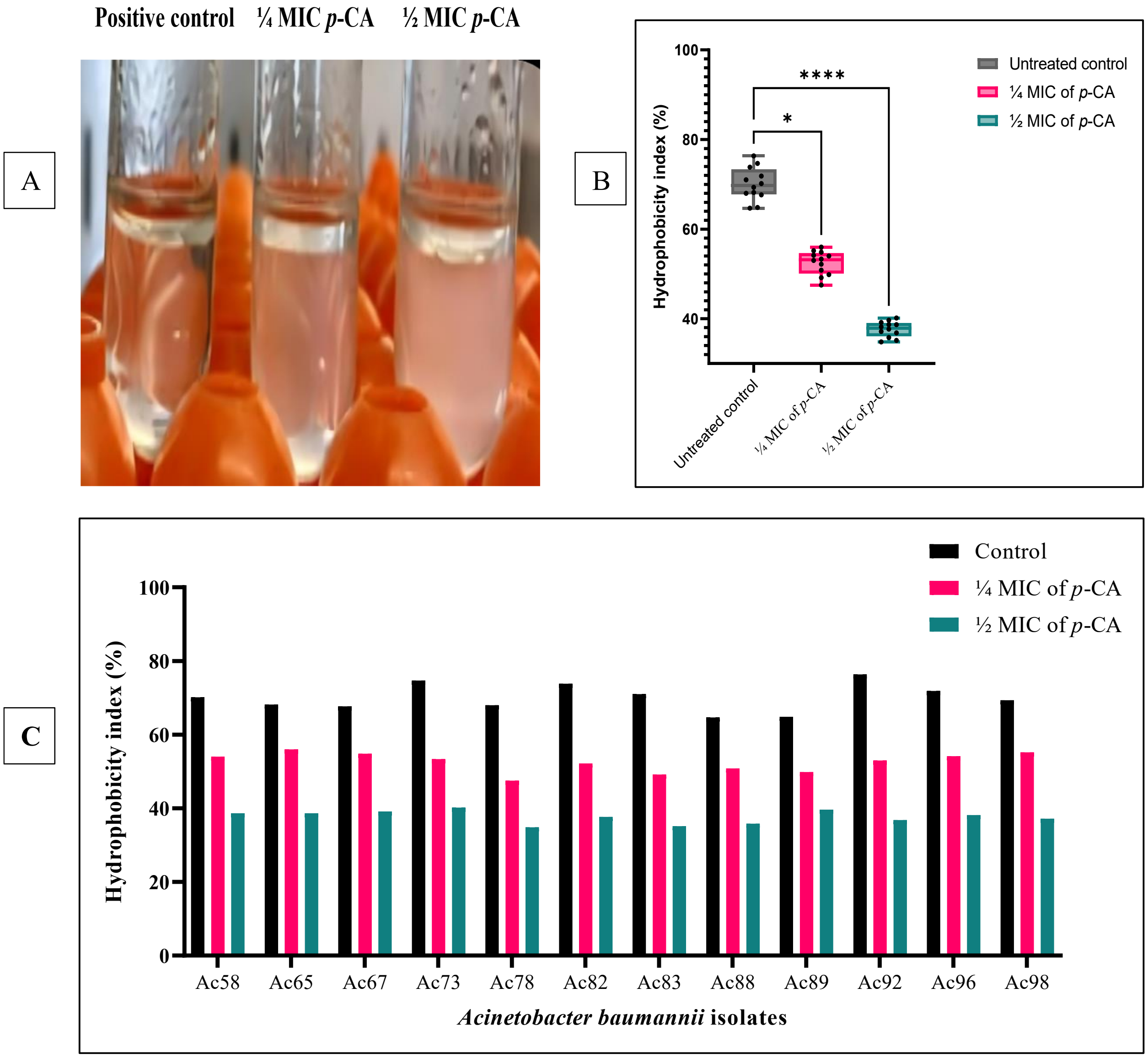

Figure S9: Effect of ¼ MIC and ½ MIC of p-coumaric acid (*p*-CA) on the cell surface hydrophobicity of strong biofilm-forming IXDRAb isolates (*n* = 12), assessed via the microbial adhesion to hydrocarbons assay **(A)** Representative images showing differences in the turbidity of aquous phase. **(B)** Box-and-whisker plots display the distribution of hydrophobicity index percentages across the tested isolates treated with ¼ MIC or ½ MIC of *p*-CA compared to untreated controls. Box boundaries represent the 25th and 75th percentiles (interquartile range), horizontal lines within boxes indicate median values, and whiskers extend to minimum and maximum observations. Individual data points (black dots) are overlaid to illustrate the actual distribution of hydrophobicity index percentages among the tested isolates. Statistical analysis was performed using one-way ANOVA followed by Dunnett's multiple comparison test $*p < 0.05$; $****p < 0.0001$ vs. untreated control. **(C)** Bars represent mean hydrophobicity index percentages for each untreated strong biofilm-forming isolate and after treatment with ¼ MIC or ½ MIC of *p*-CA.

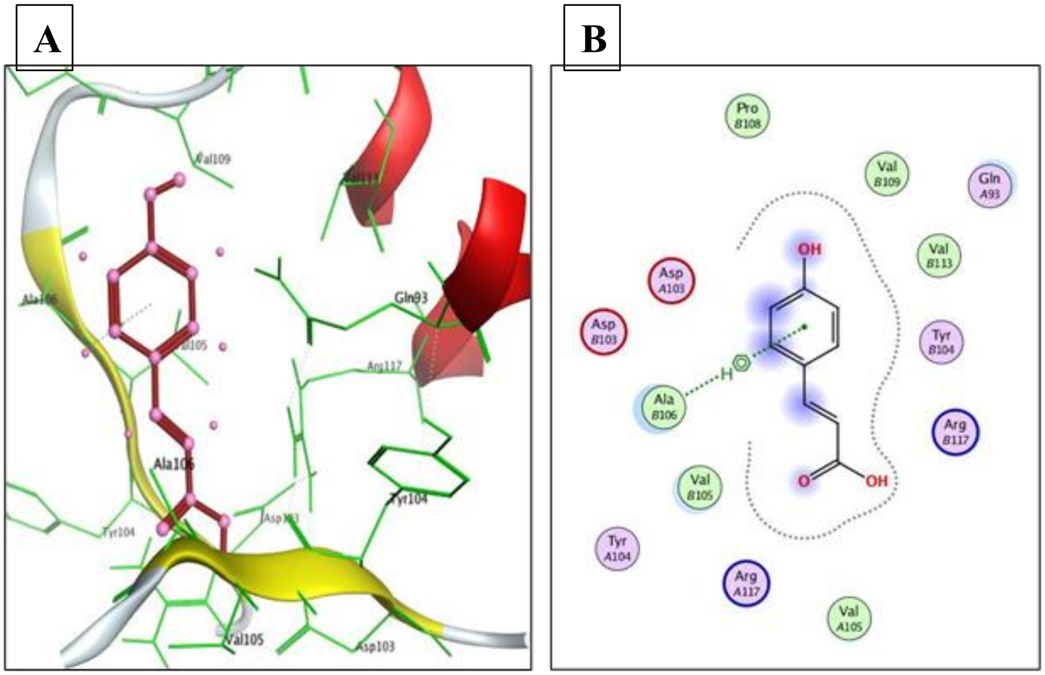

Figure S10: *In silico* analysis of the binding of BfmR protein of *Acinetobacter baumannii* to p-coumaric acid (*p*-CA). (**A**) The three-dimensional binding mode of *p*-CA with BfmR protein (the *p*-CA is colored in red and pink). (**B**) The two-dimensional binding mode of *p*-CA with BfmR protein

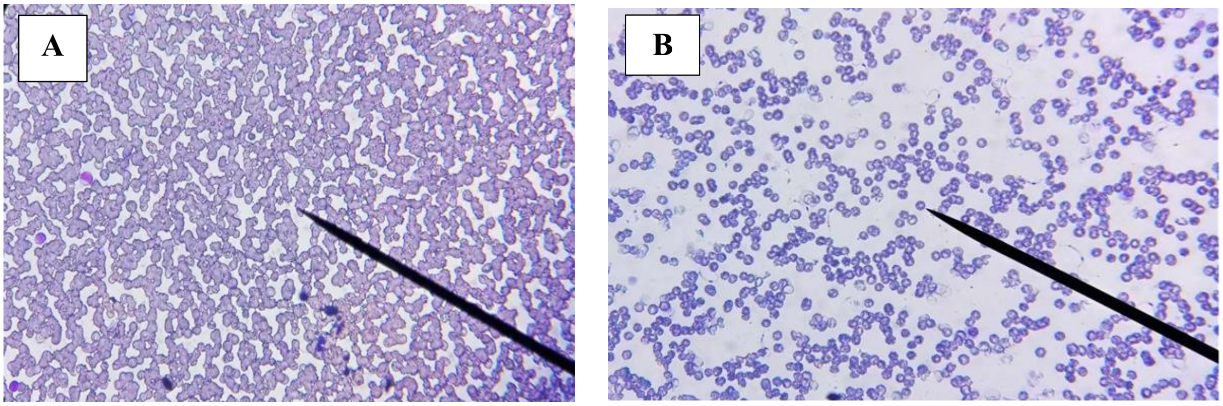

Figure S11: Microscopic examination of Giemsa-stained peripheral blood films from untreated (**A**) and cyclophosphamide-treated (**B**) rats. The marked reduction in total blood cell density observed in the treated group demonstrates effective induction of an immunocompromised state.

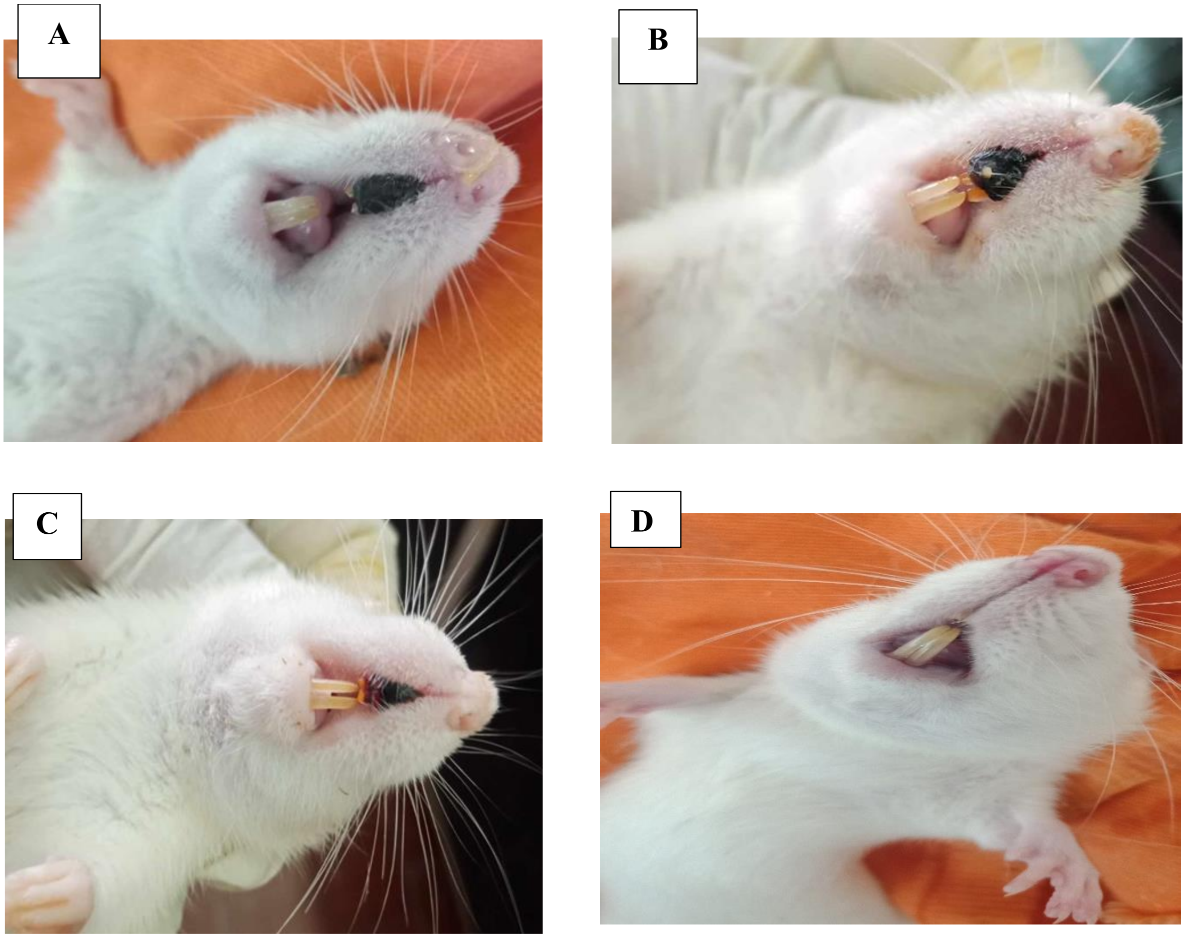

Figure S12: Photographs of representative rats from each group on day 3 to show the difference in the severity of blackened tissue appearance as clinical symptoms, due to the necrotizing ability of multidrug *Acinetobacter baumannii* among the tested groups, (**A**) represented group 2 of the positive control, (**B**) was group 3 of the imipenem- treated rats, (**C**) group 4 of the p-coumaric acid- treated rats, and (**D**) group 5 of the double- treated rats.

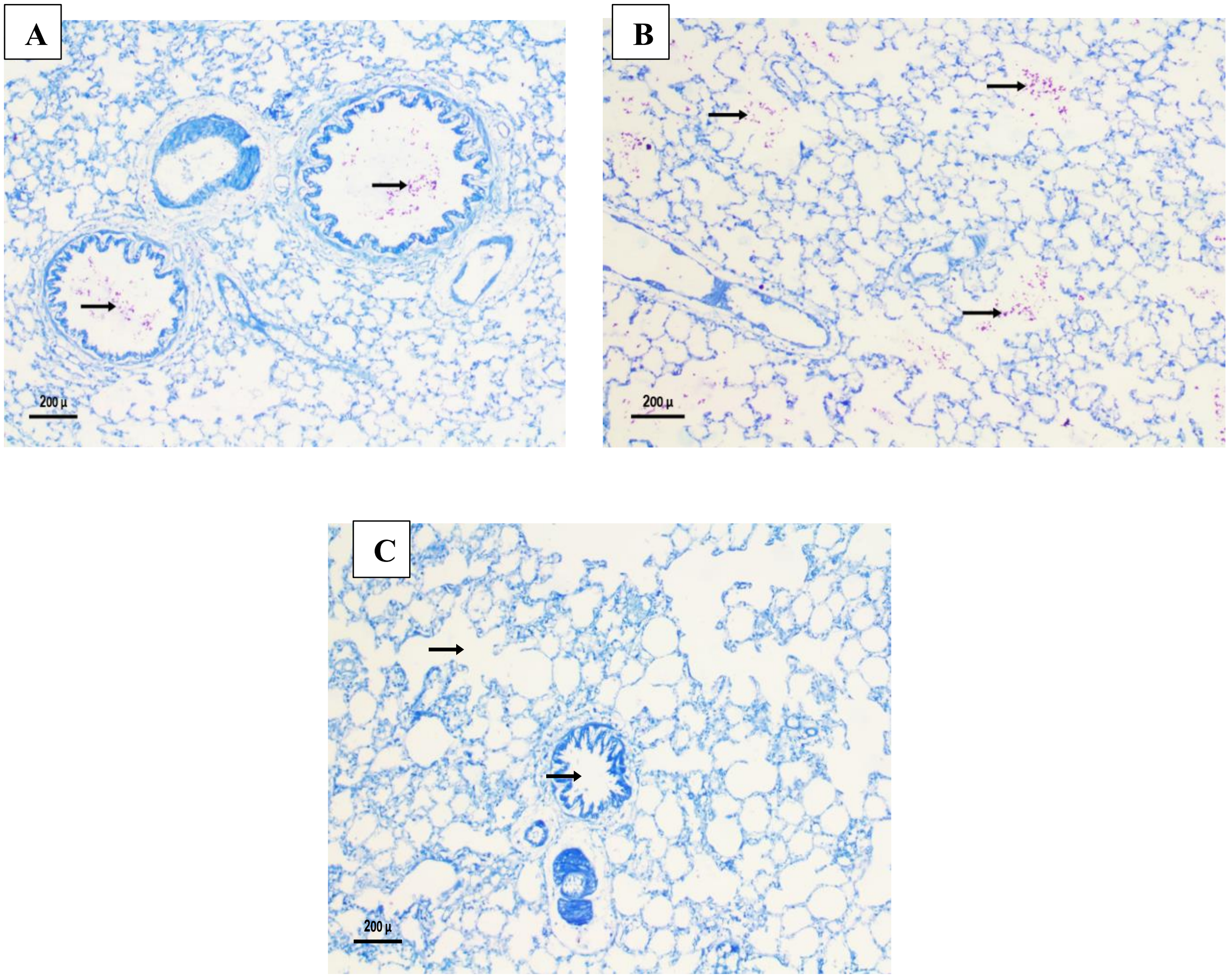

Figure S13: Histopathological examination of Giemsa-stained lung tissues revealed abundant purple-stained *Acinetobacter baumannii* cells in the lumens of bronchioles (thin arrows) (**A**) and alveoli (thin arrows) (**B**) in the infection-positive control group. In contrast, panel (**C**) shows the lungs of rat treated with the dual combination rescue therapy, displaying normal histoarchitecture with no detectable bacterial presence in bronchiolar or alveolar spaces (Magnification ×100).

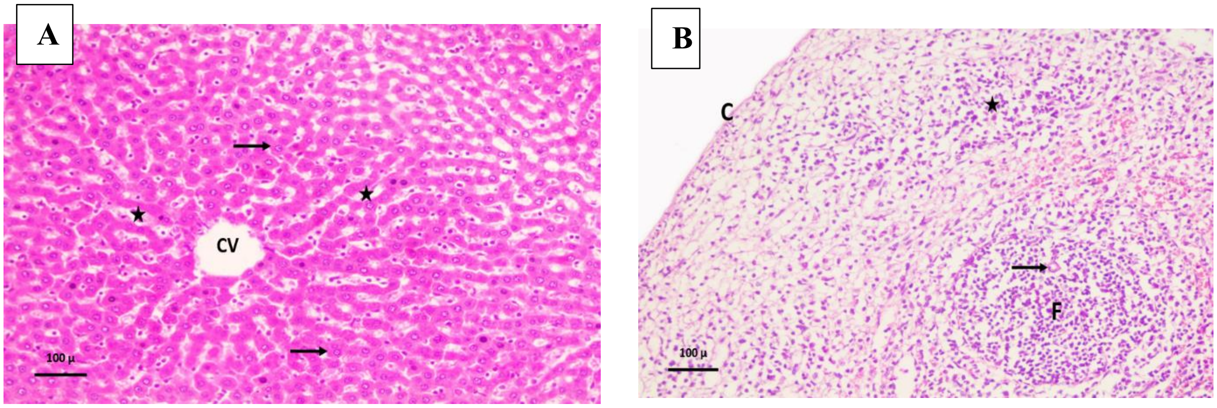

Figure S14: Representative photomicrographs confirming the absence of pathological changes in control organs (**A**) Liver section (Group 5) demonstrating preserved hepatic architecture: Central vein (CV) with radially arranged hepatocyte cords, showing characteristic polyhedral hepatocytes with central vesicular nuclei (→) and eosinophilic cytoplasm, separated by sinusoidal spaces (*). (H&E, Magnification ×200) (**B**) Splenic section (Group 5) exhibiting normal microstructure: Intact capsule (C), white pulp with lymphoid follicles (F) containing central arterioles (→), and red pulp (R) with sinusoidal vasculature. (H&E, Magnification ×200)

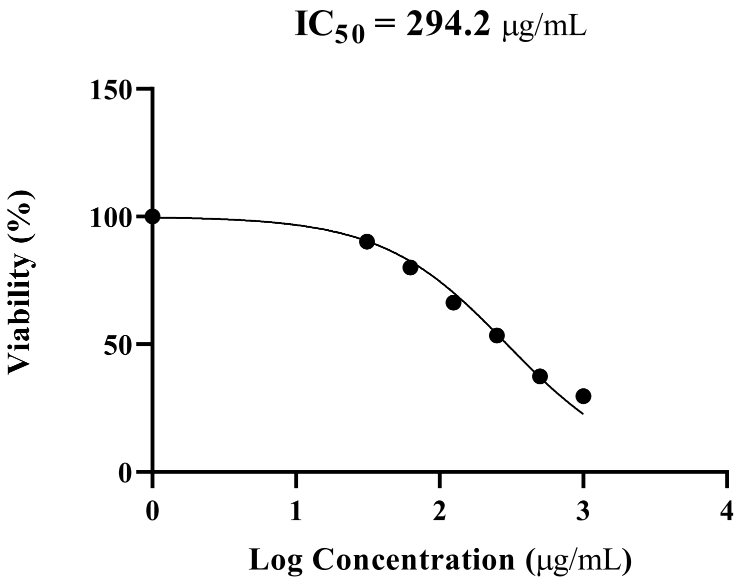

Figure S15: Dose-response curve illustrating the cytotoxic effect of p-coumaric acid on human skin fibroblast normal cells using the MTT assay. The IC_{50} value was calculated as 294.2 µg/mL, indicating the concentration required to inhibit 50% of cell viability.

References

1. Stepanović S, Vuković D, Hola V, Di Bonaventura G, Djukić S, Ćirković I, et al. Quantification of biofilm in microtiter plates: Overview of testing conditions and practical recommendations for assessment of biofilm production by staphylococci. APMIS. Blackwell Munksgaard; 2007;115:891–9. https://doi.org/10.1111/j.1600-0463.2007.apm_630.x

2. Sherif MM, Elkhatib WF, Khalaf WS, Elleboudy NS, Abdelaziz NA. Multidrug Resistant Acinetobacter baumannii Biofilms: Evaluation of Phenotypic–Genotypic Association and Susceptibility to Cinnamic and Gallic Acids. Front Microbiol. 2021;12. https://doi.org/10.3389/fmicb.2021.716627

3. Famuyide IM, Aro AO, Fasina FO, Eloff JN, McGaw LJ. Antibacterial and antibiofilm activity of acetone leaf extracts of nine under-investigated south African Eugenia and Syzygium (Myrtaceae) species and their selectivity indices. BMC Complement Altern Med. 2019;19:141. https://doi.org/10.1186/s12906-019-2547-z

4. Kırmusaoğlu S. The Methods for Detection of Biofilm and Screening Antibiofilm Activity of Agents. Antimicrobials, Antibiotic Resistance, Antibiofilm Strategies and Activity Methods. IntechOpen; 2019. https://doi.org/10.5772/intechopen.84411

5. Mufti IU, Gondal A, Kiyani KM, Mufti SM, Shahid R, Ihsan A, et al. Microstructural, physico-chemical, antibacterial and antibiofilm efficacy of imipenem loaded chitosan nano-carrier systems to eradicate multidrug resistant Acinetobacter baumannii. Mater Today Commun. 2023;35:105874. https://doi.org/10.1016/j.mtcomm.2023.105874

6. Wang S, Ma C, Long J, Cheng P, Zhang Y, Peng L, et al. Impact of CRAMP-34 on Pseudomonas aeruginosa biofilms and extracellular metabolites. Front Cell Infect Microbiol. 2023;13. https://doi.org/10.3389/fcimb.2023.1295311

7. Ghasemi M, Turnbull T, Sebastian S, Kempson I. The MTT Assay: Utility, Limitations, Pitfalls, and Interpretation in Bulk and Single-Cell Analysis. Int J Mol Sci. 2021;22:12827. https://doi.org/10.3390/ijms222312827

8. Sankar Ganesh P, Ravishankar Rai V. Attenuation of quorum-sensing-dependent virulence factors and biofilm formation by medicinal plants against antibiotic resistant Pseudomonas aeruginosa. J Tradit Complement Med. 2018;8:170–7. https://doi.org/10.1016/j.jtcme.2017.05.008

9. Rashiya N, Padmini N, Ajilda AAK, Prabakaran P, Durgadevi R, Veera Ravi A, et al. Inhibition of biofilm formation and quorum sensing mediated virulence in Pseudomonas aeruginosa by marine sponge symbiont Brevibacterium casei strain Alu 1. Microb Pathog. 2021;150:104693. https://doi.org/10.1016/j.micpath.2020.104693

10. Gowrishankar S, Kamaladevi A, Balamurugan K, Pandian SK. *In Vitro* and *In Vivo* Biofilm Characterization of Methicillin-Resistant *Staphylococcus aureus* from Patients Associated with Pharyngitis Infection. Biomed Res Int. 2016;2016:1–14. https://doi.org/10.1155/2016/1289157

11. Mu Y, Zeng H, Chen W. Quercetin Inhibits Biofilm Formation by Decreasing the Production of EPS and Altering the Composition of EPS in Staphylococcus epidermidis. Front Microbiol. 2021;12. https://doi.org/10.3389/fmicb.2021.631058

12. Awadelkareem AM, Al-Shammari E, Elkhalifa AO, Adnan M, Siddiqui AJ, Mahmood D, et al. Anti-Adhesion and Antibiofilm Activity of Eruca sativa Miller Extract Targeting Cell Adhesion Proteins of Food-Borne Bacteria as a Potential Mechanism: Combined In Vitro-In Silico Approach. Plants. 2022;11:610. https://doi.org/10.3390/plants11050610

13. Martínez Chamás J, Isla MI, Zampini IC. Antibacterial and Antibiofilm Activity of Different Species of Fabiana sp. Extract Obtained via Maceration and Ultrasound-Assisted Extraction against Staphylococcus epidermidis. Plants. 2023;12:1830. https://doi.org/10.3390/plants12091830

14. Karunanidhi A, Ghaznavi-Rad E, Hamat RA, Pichika MR, Lung LTT, Mohd Fauzi F, et al. Antibacterial and Antibiofilm Activities of Nonpolar Extracts of *Allium stipitatum* Regel. against Multidrug Resistant Bacteria. Biomed Res Int. 2018;2018:1–13. https://doi.org/10.1155/2018/9845075

15. Upmanyu K, Rizwanul Haq QMohd, Singh R. Antibacterial and Antibiofilm Properties of the Alexidine Dihydrochloride (MMV396785) against Acinetobacter baumannii. Antibiotics. 2023;12:1155. https://doi.org/10.3390/antibiotics12071155

16. Yang C-H, Su P-W, Moi S-H, Chuang L-Y. Biofilm Formation in Acinetobacter Baumannii: Genotype-Phenotype Correlation. Molecules. 2019;24:1849. https://doi.org/10.3390/molecules24101849

17. Zeighami H, Valadkhani F, Shapouri R, Samadi E, Haghi F. Virulence characteristics of multidrug resistant biofilm forming Acinetobacter baumannii isolated from intensive care unit patients. BMC Infect Dis. 2019;19:629. https://doi.org/10.1186/s12879-019-4272-0

18. Lee H-W, Koh YM, Kim J, Lee J-C, Lee Y-C, Seol S-Y, et al. Capacity of multidrug-resistant clinical isolates of Acinetobacter baumannii to form biofilm and adhere to epithelial cell surfaces. Clinical Microbiology and Infection. 2008;14:49–54. https://doi.org/10.1111/j.1469-0691.2007.01842.x

19. Livak KJ, Schmittgen TD. Analysis of Relative Gene Expression Data Using Real-Time Quantitative PCR and the $2^{-\Delta\Delta CT}$ Method. Methods. 2001;25:402–8. https://doi.org/10.1006/meth.2001.1262

20. Wójciak-Kosior M, Paduch R, Matysik-Woźniak A, Niedziela P, Donica H. The effect of ursolic and oleanolic acids on human skin fibroblast cells. Folia Histochem Cytobiol. 2012;49:664–9. https://doi.org/10.5603/FHC.2011.0050
